# FGF13 Alleviates the Aging Process in Mice by Regulating Astrocyte Function in the Medial Prefrontal Cortex via Phafin2 Signaling Pathway

**DOI:** 10.1111/acel.70784

**Published:** 2026-10-25

**Authors:** Rui Zheng, Zheng Tu, Yang Fang, Caixiang Zhuang, Yuchao Zhang, Yibo Ying, Zhiyi Feng, Ziyang Jin, Yanyan Zheng, Zhouguang Wang

**Affiliations:** ^1^ Wenzhou Third Clinical Institute Affiliated to Wenzhou Medical University, Wenzhou People's Hospital Wenzhou China; ^2^ State Key Laboratory of Macromolecular Drugs and Large‐Scale Preparation, School of Pharmaceutical Science Wenzhou Medical University Wenzhou China; ^3^ Department of Laboratory Medicine The Third Affiliated Hospital of Zhengzhou University Zhengzhou Henan China; ^4^ The First Affiliated Hospital of Wenzhou Medical University Wenzhou China; ^5^ Oujiang Laboratory (Zhejiang Lab for Regenerative Medicine, Vision and Brain Health), School of Pharmaceutical Science Wenzhou Medical University Wenzhou China

**Keywords:** aging, astrocyte, brain, central nervous system, FGF13

## Abstract

Aging is an intrinsic biological process; specifically, brain aging leads to cognitive decline, memory loss, and numerous neurodegenerative diseases. Fibroblast growth factor 13 (FGF13) is expressed in the central nervous system (CNS) and plays a critical role in several neuropathological conditions; however, the specific role of FGF13 in brain aging remains unclear. In this study, screening of public gene expression databases revealed that *FGF13* is significantly downregulated in the brain tissues of aging mice and macaques, particularly within the medial prefrontal cortex (mPFC) and astrocytes. Specific knockout of *FGF13* in cortical astrocytes accelerates the aging phenotype and impairs neurological function in mice. Conversely, overexpression of FGF13 effectively delays the aging process and ameliorates aging‐induced cognitive impairment. RNA‐seq analysis indicated that FGF13 primarily influences astrocyte‐mediated inflammatory responses and cellular autophagy. Mechanistically, FGF13 targets and binds to Phafin2, promoting autophagy in astrocytes. Homeostatic astrocytes not only ameliorate neuronal damage but also reduce the release of inflammatory factors by microglia, playing a crucial role in mitigating the aging process in mice. In summary, FGF13 improves the cerebral microenvironment by regulating astrocyte function, ultimately exerting an anti‐aging effect. These findings provide a theoretical basis for neuroscience and aging research, offering new insights into age‐related genetic phenotypes in the brain and body, as well as the interplay between aging and various diseases.

## Graphical abstract

FGF13 is significantly downregulated in the aging brain, particularly in cortical astrocytes within the medial prefrontal cortex. Genetic knockdown of FGF13 in astrocytes accelerates aging phenotypes and impairs neurological function in mice, while its overexpression effectively delays cognitive decline. Mechanistically, FGF13 binds to Phafin2 to promote autophagy in astrocytes, which not only ameliorates neuronal damage but also reduces microglia‐derived inflammatory factors, thereby improving the cerebral microenvironment. Together, these findings establish FGF13 as a key regulator of astrocyte homeostasis that exerts anti‐aging effects, offering new insights into age‐related brain dysfunction and potential therapeutic strategies.

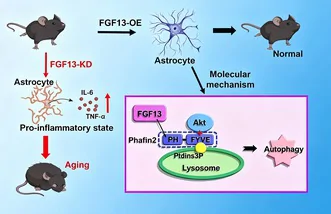

## 1 Introduction

Aging is an intrinsic biological process characterized by the gradual deterioration of the physiological functions of cells, tissues, and organs over time (Blinkouskaya et al. 2021; Haydont et al. 2019). This process increases susceptibility to age‐related diseases and ultimately leads to death. The hallmarks of aging include cellular senescence, genomic instability, epigenetic alterations, loss of proteostasis, stem cell exhaustion, chronic inflammation, and altered intercellular communication (Hou et al. 2019; Campisi et al. 2019). Notably, the time‐dependent functional decline of the brain is a critical factor contributing to the onset of neurodegenerative diseases (Gooijers et al. 2024). Brains of patients with neurodegenerative disorders frequently exhibit distinct features of aging, primarily including glia‐mediated chronic inflammation, neural network dysfunction, as well as neuronal senescence and apoptosis (Lee and Kim 2022; Sahu et al. 2022). Although these hallmarks provide a fundamental understanding of brain aging, our comprehension of how the brain—a complex and highly heterogeneous multicellular tissue—ages remains at a nascent stage. The rapid development of current single‐cell sequencing technologies has provided a vital foundation for revealing cellular characteristics of aging at the cell‐type level (Allen et al. 2023). While cellular senescence is generally considered irreversible, research indicates that senescence in specific cell types may be dynamic. Therefore, characterizing the molecular and cellular changes associated with aging will not only supplement our insight into this complex process but also facilitate the exploration of methods to slow or reverse it, thereby aiding in the prevention or treatment of age‐related pathologies.

Fibroblast Growth Factors (FGFs) serve as a crucial class of signaling molecules that play key roles in biological processes such as cell migration, proliferation, differentiation, metabolic regulation, and the maintenance of neural function (Ternier et al. 2025; Bollenbecker et al. 2023). They are also widely involved in pathophysiological processes, including development, metabolic disorders, neurodegenerative diseases, and tumorigenesis (Tan et al. 2023). The FGF family consists of structurally related proteins that can be categorized into secreted (FGF1‐10 and FGF15‐23) and non‐secreted (FGF11‐14) types. Non‐secreted FGFs, also known as FGF Homologous Factors (FHFs), share genomic sequence and structural similarities with other FGF family members but exhibit predominantly high expression in the central nervous system (CNS), particularly within neurons (Alam et al. 2022; Mahling et al. 2021). FGF13 is a prominent member of the non‐secreted FGF subfamily; its biological functions are independent of the fibroblast growth factor receptor (FGFR) signaling pathway, allowing it to exhibit a broader range of physiological regulatory roles (Naderi et al. 2025). Research has found that FGF13 participates in regulating biological processes such as neuronal excitability, polarization, and neurogenesis, and is involved in numerous pathophysiological conditions, including intellectual developmental disorders, diabetes, and cancer (Song et al. 2025; Sun et al. 2023). However, the role of FGF13 in the regulation of aging has not yet been reported.

Accumulating evidence suggests that FGF13 plays a significant role in neuroinflammatory responses and can effectively ameliorate glial‐neuronal functional impairment in Parkinson's disease (PD) (Song et al. 2025). Neurons serve as the fundamental working units of the brain, and their activity directly influences the brain's physiological and pathological states. Glial cells (primarily astrocytes, microglia, and oligodendrocytes) are dominant in number; these cells actively support neuronal function and act as indispensable participants in neuronal injury under pathological conditions (Kwon and Koh 2020). The memory and cognitive deficits resulting from neuronal damage and apoptosis during aging are major factors leading to neurodegenerative diseases (Bhat and Dhaneshwar 2024). Since astrocytes can effectively process and degrade waste products released by neuronal abnormalities, the interaction between neurons and glia plays a central role in maintaining brain physiology and promoting neuropathology (Acioglu et al. 2021). Therefore, investigating how FGF13 influences the nervous system is of great significance for delaying and treating brain injury caused by aging.

In this study, we conducted single‐cell sequencing analyses of aging mice and aging macaques from the GEO public dataset, followed by a series of experimental investigations. The results revealed that FGF13 expression is significantly downregulated in brain astrocytes in both aging mouse and macaque models. When *FGF13* was specifically knocked out in cortical astrocytes, mice exhibited cognitive impairment and accelerated aging; conversely, overexpression of *FGF13* effectively ameliorated the aging process. Furthermore, overexpression of *FGF13* in astrocytes in vitro effectively reduced aging‐induced neuroinflammation and injury. Regarding the mechanism of action, FGF13 binds to Phafin2 to regulate cellular autophagy to improve astrocyte function. These findings provide novel insights for FGF13‐based pharmacotherapy and expand the range of potential therapeutic targets for the prevention and treatment of aging and related diseases.

## 2 Materials and Methods

### 2.1 Animal and Experimental Design

All animal‐related experiments were strictly conducted in accordance with the Animal Care Ethics Guidelines of Wenzhou Medical University and were approved by the Ethics Committee (XMSQ2025‐0259). The mice were housed in a specific pathogen‐free (SPF) environment, with room temperature maintained at 22°C ± 2°C and a 12‐h light/dark cycle. The animals had free access to sterilized SPF pellet rodent feed and sterilized water. FGF13^flox/flox^ (FGF13^fl/fl^) mice on a C57BL/6J background and GFAP‐cre mice on a C57BL/6J background were generously provided by Professor Kong from Wenzhou University. Six‐month‐old C57BL/6J mice were selected as the young group. For FGF13 knockdown (FGF13‐KD): We used FGF13^(fl/fl) mice (which harbor loxP sites flanking the FGF13 coding exons) and injected them with an AAV9 vector carrying GFAP‐Cre (pAAV‐GfaABC1D‐mCherry‐P2A‐Cre‐WPRE at a titer of 5 × 10^12^ vg/mL, supplied by OBIO Technology). The GFAP promoter drives Cre expression specifically in astrocytes, thereby achieving astrocyte‐specific conditional knockout of FGF13 in the injected region. This does not involve breeding FGF13^fl/fl^ with GFAP‐Cre transgenic mice. For FGF13 overexpression (FGF13‐OE): We used GFAP‐Cre transgenic mice (which constitutively express Cre recombinase under the GFAP promoter) and injected them with an AAV9 vector carrying a Cre‐dependent FGF13 overexpression cassette (pAAV‐CMV‐DIO‐Fgf13‐3xFLAG‐T2A‐EGFP‐WPRE at a titer of 5 × 10^12^ vg/mL, supplied by OBIO Technology, and the reference sequence for *FGF13* was NM_010200.3). In this configuration, Cre‐mediated recombination removes the loxP‐flanked STOP cassette, allowing FGF13 overexpression specifically in astrocytes. The “Aging” control group consisted of two separate cohorts: for the FGF13‐KD group (FGF13^fl/fl^ mice injected with AAV‐GFAP‐Cre), the corresponding aging controls were FGF13^fl/fl^ mice; and for the FGF13‐OE group (GFAP‐Cre mice injected with AAV‐DIO‐FGF13), the corresponding aging controls were GFAP‐Cre mice. All of these groups were composed of 16‐month‐old mice, and both the starting age (16 months) and the final age at sacrifice (20–21 months), thereby eliminating any ambiguity regarding the age‐related phenotype. Each group consists of 10 to 12 mice. To eliminate the influence of mouse gender, each group has an equal number of male and female mice.

### 2.2 Stereotaxic Micro‐Injection

As described in previous studies (Zheng et al. 2025), mice were anesthetized with isoflurane (3% for induction, 1.5% for maintenance) and then secured in a digital stereotaxic instrument (RWD Life Science Co. Ltd., China) for viral injection. The scalp was incised, the skull was scrubbed with iodine and rinsed with saline. Unless otherwise specified, all coordinate measurements for different sites were relative to bregma. The aforementioned AAVs were injected into the mPFC region (AP +1.40 mm, ML ±1.90 mm, DV –1.50 mm). The injection volume was 200 nL, with the flow rate controlled at 40–50 nL/min. Following injection, the needle was left in place for approximately 5 min before being slowly withdrawn after sealing the needle track with saline. After suturing the incision, the mice were transferred to a heating pad until they regained consciousness.

### 2.3 Cell Culture and Transfection

C6 cells (cat. no. TCR1) were purchased from the Cell Bank of the Chinese Academy of Sciences and cultured in DMEM (Gibco) containing 4.5 g/L glucose, supplemented with 10% fetal bovine serum and 1% penicillin/streptomycin (Gibco), at 37°C in a 5% CO_2_ atmosphere. After stable cell growth was achieved, C6 cells were seeded into 6‐well plates (10^5^ cells/pre well) for infection and transfection, and cultured overnight until they reached 30%–40% confluence. FGF13 knockdown was performed using FGF13‐specific short hairpin RNA (shRNA) and its negative control as previously described. The shFGF13 sequence information was as follows: 5′‐caccgcacttacactctgtttaaccttcaagagagttaacagtgtaagtgcttttg‐3′ and 5′‐gatccaaaaaagcacttacactctgttaacctctgaaggttaaacagtgtaagtgc‐3′. The negative control sequence information was as follows: 5′‐caccgttctccgaacgtgtcacgtcaagagattacgtgacacgttcggagaattttttg‐3′ and 5′‐gatccaaaaaattctccgaacgtgtcacgtaatctcttgacgtgacacgttcggagaac‐3′. The lentiviral vectors were both constructed in the pLKO.1‐EGFP‐Puro backbone (Chemshine, Shanghai, China). The lentivirus titer was 6 × 10^8^ TU/mL. For FGF13 overexpression, the FGF13 coding sequence (NM_010200.3) was cloned into the pcSLenti‐CMV‐3xFlag‐PGK‐Puro‐WPRE3 backbone (OBiO, Shanghai, China), and the empty vector (without FGF13) served as the control. The overexpression lentivirus titer was 8 × 10^8^ TU/mL. After lentiviral infection at an MOI of 10, the cells were incubated with 6 μg/mL polybrene for 12 h to enhance infection efficiency. The supernatant was then replaced with fresh culture medium. When cell confluence reached 80%, 3 μg/mL puromycin was added to the culture medium to eliminate uninfected cells. Once stably transfected cells emerged, the puromycin concentration in the medium was reduced to 1 μg/mL to continue the selection of infected cells. The resulting stable cell lines were designated as sh‐FGF13 and AAV‐FGF13, respectively. PC12 cells (SCSP‐5259) were purchased from the Cell Bank of the Chinese Academy of Sciences and cultured in RPMI 1640 medium (Gibco), supplemented with 10% fetal bovine serum and 1% penicillin/streptomycin (Gibco), at 37°C in a 5% CO2 atmosphere. BV2 cells (SCSP‐5208) were purchased from the Cell Bank of the Chinese Academy of Sciences and cultured in DMEM (Gibco) containing 4.5 g/L glucose, supplemented with 10% fetal bovine serum and 1% penicillin/streptomycin (Gibco), at 37°C in a 5% CO2 atmosphere.

### 2.4 Primary Astrocyte Culture

Primary astrocytes were isolated from the medial prefrontal cortex (mPFC) of postnatal day 0–2 (P0–P2) C57BL/6 mouse pups, as previously described with minor modifications (Zheng et al. 2026). Briefly, brain tissues were carefully dissected, the meninges were removed, and the mPFC was minced into small pieces (~1 mm^3^) using sterile surgical scissors. Tissue fragments were then digested with 0.25% papain (Worthington, USA) in Hanks' Balanced Salt Solution (HBSS) at 37°C for 15 min with intermittent gentle agitation. Following enzymatic digestion, the tissue was mechanically triturated using a fire‐polished glass Pasteur pipette to obtain a single‐cell suspension. The cell suspension was filtered through a 70 μm cell strainer (Falcon, USA) to remove undigested debris, and the filtrate was collected by centrifugation at 300 × g for 5 min. The resulting cell pellet was resuspended in DMEM/F12 (Gibco, USA) supplemented with 20% fetal bovine serum (FBS, Gibco, USA), 100 U/mL penicillin, and 100 μg/mL streptomycin (Gibco, USA). Cells were plated onto poly‐D‐lysine (10 μg/mL; Sigma‐Aldrich, USA) coated T75 culture flasks and maintained in a humidified incubator at 37°C with 5% CO_2_. The culture medium was replaced every 3 days.

After 7–10 days in culture, when the cells reached approximately 80%–90% confluence, astrocytes were purified by differential trypsinization to remove loosely adherent microglia and oligodendrocyte precursor cells. Briefly, flasks were incubated with 0.25% trypsin–EDTA (Gibco, USA) for 1–2 min at 37°C. Under these conditions, astrocytes remain adherent while microglia and OPCs detach more rapidly. The detached cells were aspirated, and fresh trypsin–EDTA was added for an additional 5–7 min at 37°C to detach the remaining astrocytes. The astrocyte‐enriched population was collected, pelleted, and re‐plated in fresh culture medium. Cell purity was assessed by immunocytochemical staining against glial fibrillary acidic protein (GFAP; anti‐GFAP antibody, Sigma‐Aldrich, USA) and confirmed to be ≥ 95% GFAP‐positive. Astrocytes at passages 2–3 were used for all subsequent experiments to maintain phenotypic stability and minimize culture‐induced alterations. Lentiviral infection is consistent with the transfection method of C6 cells.

### 2.5 Behavioral Analysis

To evaluate spatial learning, the Morris Water Maze (MWM) test was conducted as previously described (Dinel et al. 2020). The animals were tested in a circular water tank, 1.2 m in diameter, located in a room with extra‐maze visual cues. A platform (8 cm in diameter) was submerged 1 cm below the water surface, and the water temperature was maintained between 23°C and 25°C throughout testing. Non‐toxic white tempera was added to the water to render it opaque and hide the platform. During the acquisition phase, each animal underwent four trials per day for 5 consecutive days. The location of the hidden platform remained in the same quadrant for all animals; however, the starting positions were pseudo‐randomly selected. Each animal was allowed 60 s to locate the hidden platform. If a mouse failed to find the platform within 60 s, it was gently guided to the platform location by nudging it forward from the base of its tail and maneuvering it toward the platform. The animals were allowed to remain on the platform for 10 s to encode spatial cues. The mouse was then returned to a resting cage on a heating pad for 25 s before the next trial. A probe trial was conducted 24 h after the final acquisition trial. During the probe trial, the platform was removed, and the mouse was allowed to swim freely for 60 s. All trials were recorded, and the data were analyzed using ANY‐maze software (Stoelting Co., USA). The dependent variables analyzed were the latency (in seconds) to reach the platform during the acquisition trials, the number of platform crossings, the latency (in seconds) to the first crossing of the platform location, and the swimming speed (cm/s) during the probe trial.

The Open Field Test (OFT) was conducted in a rectangular chamber (40 × 40 × 30 cm) constructed from gray polyvinyl chloride and illuminated by a 25 W halogen lamp positioned 180 cm above the arena. Each mouse was gently placed in the center of the arena and allowed to explore freely for 5 min. The movement trajectories were recorded, and locomotor activity was analyzed using EthoVision 7.0 software.

Mice were acclimatized to the testing environment for 1 h prior to the experiment of Elevated Plus Maze (EPM) test. At the start of the test, each mouse was gently placed on the central platform (5 × 5 cm) of the elevated plus maze and allowed to freely explore the two opposing open arms and the two opposing enclosed arms for 5 min. The duration of time spent in the open and closed arms was tracked and recorded using a video tracking system (EthoVision 7.0).

### 2.6 Immunohistochemistry and Immunocytochemistry

For immunohistochemistry (IHC) on brain sections, brain tissues were fixed in 4% paraformaldehyde (PFA) and subsequently dehydrated in 20% and 30% sucrose dissolved in PBS for 2 days. After embedding the tissues in OCT compound, 20 μm‐thick coronal sections were obtained using a cryostat (Thermo). The brain sections were briefly washed three times with PBS. All sections were then blocked with 5% BSA and 0.3% Triton X‐100 in PBS (5% BSA/PBST), followed by incubation with primary antibodies at 4°C overnight. Subsequently, the sections were incubated with secondary antibodies at room temperature for 1 hour. After three washes with PBS, the fluorescently labeled sections were visualized using a confocal laser scanning microscope (Carl Zeiss). The fluorescence area in each image was quantified using ImageJ software. For immunocytochemical staining, neural cells were rinsed with PBS and fixed with 4% PFA for 20 min. Cell staining was performed using the same protocol as described for the immunohistochemistry of brain sections. Detailed information regarding the primary antibodies used is provided in Table S1.

### 2.7 Western Blotting

Brain tissues (Cortical tissues containing the mPFC injection site were collected for Western blot analysis) or cell samples were homogenized in a detergent‐based lysis buffer (50 mM Tris, pH 7.4, 150 mM NaCl, 1% Triton X‐100, 1% sodium deoxycholate, 0.1% SDS, and 1 mM PMSF). The homogenates were centrifuged at 16,000 × g for 30 min at 4°C. The resulting supernatants were collected, and protein concentration was quantified using a Microplate BCA Protein Assay Kit (Thermo Scientific). Equal amounts of protein samples were separated by 10% SDS‐PAGE and subsequently transferred onto polyvinylidene fluoride (PVDF) membranes. The membranes were blocked with 5% bovine serum albumin (BSA, Sigma‐Aldrich, B2064) in Tris‐buffered saline containing 0.1% Tween 20 (TBST). Following blocking, the membranes were incubated with primary antibodies at 4°C overnight. After incubation, the membranes were washed three times for 15 min each with TBST and then incubated with HRP‐conjugated goat anti‐mouse (ab205719, Abcam) or HRP‐conjugated goat anti‐rabbit (ab205718, Abcam) secondary antibodies at room temperature for 1 h. Antibody binding was detected using a chemiluminescent HRP substrate (WBKLS0500, Millipore). Protein bands were visualized using an imaging system (GE, Amersham Imager 680) and quantified with ImageJ software. All sample values were normalized to GAPDH, β‐actin, or β‐tubulin. Detailed information regarding the primary antibodies used is provided in Table S1.

### 2.8 Senescence‐Associated β‐Galactosidase (SA‐β‐Gal) Assay

The SA‐β‐gal assay was performed using a SA‐β‐gal Staining Kit (C0602, Beyotime) according to the manufacturer's instructions. Briefly, prepared tissue cryosections and neural cells were washed, fixed, and incubated with the β‐galactosidase staining solution in a dry incubator at 37°C without CO_2_. SA‐β‐gal‐positive cells exhibited blue staining and were imaged using an optical microscope (Olympus, Japan).

### 2.9 Enzyme‐Linked Immunosorbent Assay

Assays were performed according to the manufacturer's instructions for the respective ELISA kits: IL‐6 (KE10007, Proteintech), TNF‐α (KE10002, Proteintech), IFN‐γ (KE10094, Proteintech), and IL‐1β (KE10003, Proteintech). Furthermore, the glutamate content in the cell culture supernatant was measured using a Glutamate Assay Kit (ml692212, Mlbio), and the neuropeptide content was assessed using an Arginine Vasopressin Assay Kit (ml105228, Mlbio). Chemiluminescent signals were captured using an ImageQuant LAS4000 scanner (GE Healthcare, USA).

### 2.10 RNA‐Seq Analysis

Transcriptome analysis was performed via RNA‐Seq (*n* = 4) on C6 cells and FGF13‐overexpressing C6 cells following D‐gal induction treatment. RNA extraction, sequencing, and analysis were conducted as a custom service provided by Lingxi Chaowei Biological Technology Co. Ltd., using the Illumina NovaSeq 6000 platform. As our previously study (Wang et al. 2026), a total of 3 μg RNA per sample was used as an input for library preparation. Illumina Paired End Sample Prep kits (cat. no. 67700, Illumina Inc.) were used to prepare libraries according to the manufacturer's instructions. Each cDNA library was sequenced using an Illumina Hiseq 4000 (cat. no. PE150; Illumina Inc.). The raw data were processed and analyzed using the Personalbio GenesCloud online platform (genescloud.cn). Read counts for each gene were quantified using HTSeq (v0.9.1, htseq.readthedocs.io/en/release_0.9.1/) to obtain the raw expression values. Gene expression levels were then normalized to fragments per kilobase of transcript per million fragments. Differential gene expression analysis was performed using DESeq2 (v1.38.3, bioconductor.org) with screening thresholds set at|log2FoldChange|> 1 and *p* < 0.05. Bi‐directional clustering analysis of all differentially expressed genes (DEGs) was conducted using the ComplexHeatmap (v2.16.0, https://www.bioconductor.org) software package. Clustering was based on the Euclidean distance and the complete linkage method, and heatmaps were generated to visualize gene expression patterns across samples.

Gene Ontology (GO) enrichment analysis was performed to identify the biological functions associated with the DEGs. The number of DEGs enriched in each term was calculated, and GO enrichment analysis was performed on the upregulated and downregulated DEGs using ClusterProfiler (v4.6.0). *p*‐values were calculated using the hypergeometric distribution method, with a threshold of *p* < 0.05. Significantly enriched GO terms were used to determine the main biological functions, molecular functions, and cellular components associated with the DEGs. Similarly, Kyoto Encyclopedia of Genes and Genomes (KEGG) pathway enrichment analysis of the DEGs was performed using ClusterProfiler (v4.6.0) software, with a threshold of *p* < 0.05.

### 2.11 Protein–Protein Docking

For protein–protein docking, molecular docking between FGF13 and Phafin2 was performed using AutoDockTools 1.5.6 and AutoDock Vina. The crystal structures of the proteins were obtained from the AlphaFold Protein Structure Database (https://alphafold.ebi.ac.uk/). The three‐dimensional structure was FGF13 (ID: Q92913) and Phafin2 (ID: Q9H8W4). The structures were imported into PyMOL 2.4.1 to remove water molecules and initial ligands, and hydrogen atoms were added to the receptor protein. We executed separate calculations with the minimum distance between the solute and the edge of the solvation box equal to 15 Å and the spacing between grid points equal to 0.4 Å, and its charges and atom types were calculated using AutoDockTools 1.5.6. The resulting structures were saved in pdbqt format. Molecular docking and binding energy evaluation were conducted using AutoDock Vina. Re‐docking and semi‐blind docking were performed with the maximum number of poses set to 20, energy range to 10 and exhaustiveness level up to 1024. The interactions between FGF13 and the target protein Phafin2 were visualized using PyMOL 2.4.1 and Discovery Studio 2021.

### 2.12 Co‐Immunoprecipitation (Co‐IP) Assay

Cell lysates were prepared using lysis buffer supplemented with protease inhibitors. Equal amounts of protein were incubated overnight at 4°C with anti‐FGF13 or anti‐Phafin2 antibody. Protein A/G Plus‐Agarose (sc‐2003, Santa Cruz Biotechnology) was added to the samples, followed by incubation at room temperature for 4 h. The immunoprecipitates were washed three times with lysis buffer, denatured by adding 2.5× loading buffer, and boiled for 5 min. The immunoprecipitated complexes were subsequently analyzed by western blotting.

### 2.13 Transmission Electron Microscopy

Brain tissues were fixed in 2.5% glutaraldehyde in PBS buffer at room temperature for 2 h, followed by overnight fixation at 4°C. Cells were fixed in 1% osmium tetroxide at 37°C for 1 h and stained with 2% aqueous uranyl acetate for 30 min. After dehydration through a graded ethanol series (50%, 70%, 90%, and 100%) for 15 min each, the samples were transferred to absolute acetone for 20 min. The cells were infiltrated with a 1:1 mixture of absolute acetone and embedding resin for 2 h, and then embedded in pure resin. The samples were sectioned into ultrathin slices approximately 100 nm thick using a Leica UC7 ultramicrotome and imaged with a transmission electron microscope (Tecnai T10, the Netherlands).

### 2.14 Statistical Analysis

Statistical analysis was performed using GraphPad Prism 8 software. Continuous data are presented as mean ± SEM. Comparisons between two groups were conducted using an unpaired Student's *t*‐test. Differences among more than two independent groups were analyzed by one‐way analysis of variance (ANOVA). Factorial designs involving two independent variables were analyzed using Two‐way ANOVA with both factors as fixed variables, including the interaction term. When a significant interaction was detected, Bonferroni's post hoc test was applied for pairwise comparisons between groups. A *p*‐value of less than 0.05 was considered statistically significant.

## 3 Results

### 3.1 Decreased FGF13 Expression in Astrocytes Within the Medial Prefrontal Cortex of Aging Mice

To obtain a broader understanding of aging‐related changes and decipher the molecular mechanisms accompanying brain aging, transcriptomic studies in model organisms and humans have been at the forefront of experimental research. In recent years, several studies have characterized transcriptomic changes across broad brain regions during normal aging at the single‐cell level, with in‐depth targeted investigations into changes within specific regions or cell types. Therefore, to investigate the link between FGF13 and aging, we performed a screening analysis using public databases (GSE129788, CRA006617, GSE193107), and the general baseline characteristics and basic information of these datasets are summarized in Figure S1A. The results revealed a significant decrease in total *FGF13* content in the brains of aging mice (Figure 1A,B). Cell‐type specific analysis indicated no significant difference in *FGF13* expression in neurons (Figure 1C); however, *FGF13* levels were significantly reduced in astrocytes (Figure 1D), microglia (Figure 1E), and oligodendrocytes (Figure 1F). In the analysis of the proportion and quantity of each type of cell, it was found that there was no significant difference between the young group and the aging group (Figure S1D–G), these results demonstrate that the observed reduction in *FGF13* expression in aged cells is not attributable to a loss of cell numbers. In the brains of aging macaques, *FGF13* expression was significantly downregulated (Figure 1J), with marked differences observed in the medial prefrontal cortex (mPFC) and astrocytes (Figure 1K). Furthermore, *FGF13* was significantly decreased in the mouse mPFC (Figure S1). To further explore the changes in FGF13 with aging, we examined FGF13 expression in the mPFC and hippocampus of mice at different ages. The results showed that FGF13 expression in the cortex exhibited a trend of initial increase followed by a decrease with age (Figure 1L), while its expression in the hippocampus gradually declined with age (Figure 1L). These results indicate that FGF13 expression decreases as aging progresses, suggesting that FGF13 may possess significant anti‐aging potential.

**FIGURE 1:**
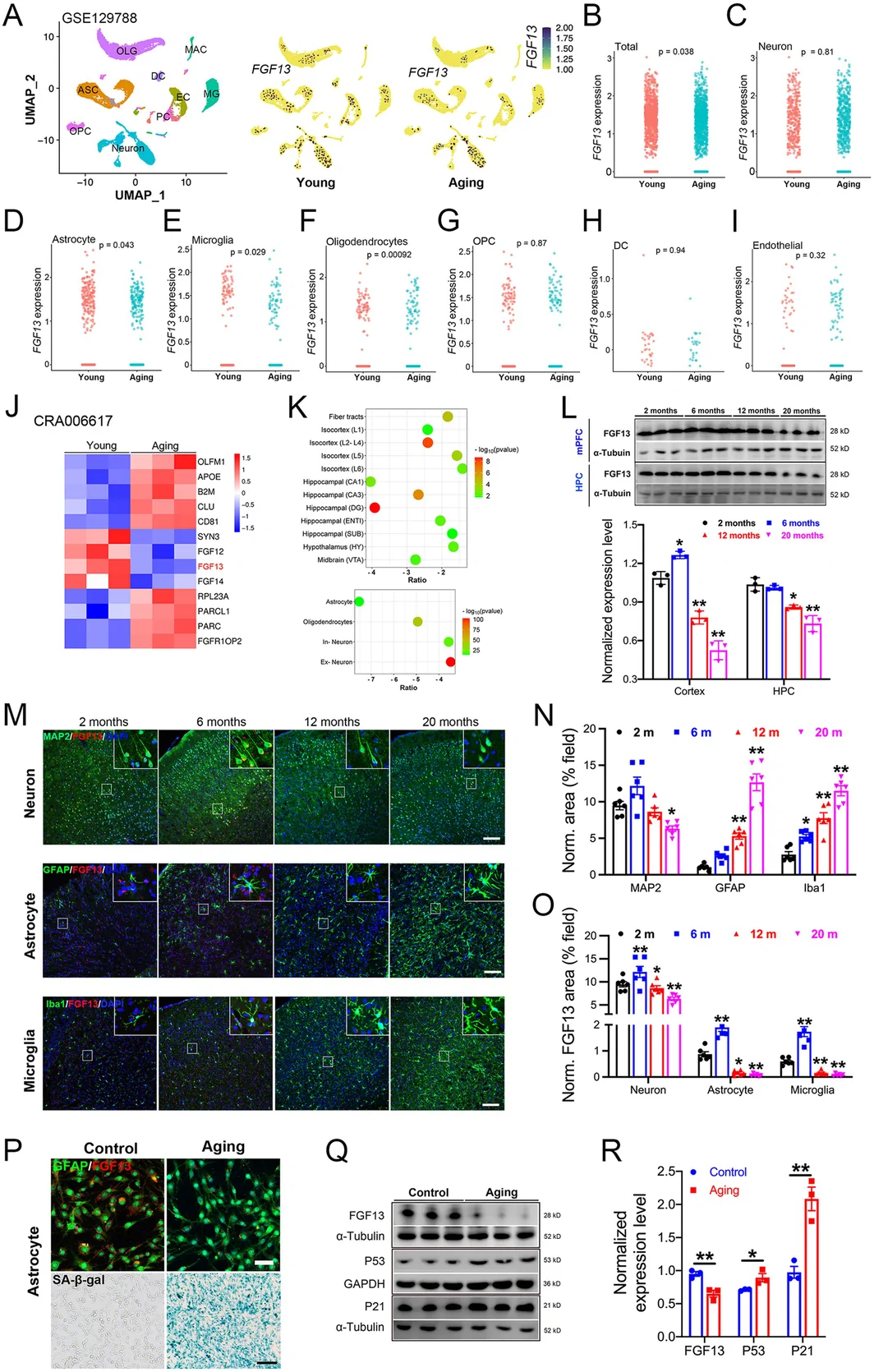
FGF13 expression decreases progressively during aging. (A) Single‐cell transcriptomic analysis of young and aged mouse brains from public database (GSE129786). UMAP plot colored by major cell types (left) and feature plot showing FGF13 expression levels across these cell types (right). (B–I) Expression changes of the FGF13 gene in various cell populations: Total cells (B), neurons (C), astrocytes (D), microglia (E), oligodendrocytes (F), oligodendrocyte precursor cells (G), dendritic cells (H), and endothelial cells (I). (J–K) Analysis of single‐cell RNA sequencing data from young and aged macaque brain tissues (public database CRA006617). Heatmap displays total FGF13 expression levels (J), and FGF13 expression levels across different brain regions and cell types (K). (L) (Top) Representative western blot of FGF13 protein expression in the mPFC and hippocampus (HPC) of mice at different ages. (Bottom) Statistical analysis of FGF13 protein expression levels, *n* = 3 per group. (M) Representative fluorescence images showing neural cells and FGF13 expression in the cortical regions of mice at different ages. Neurons (MAP2, green), astrocytes (GFAP, green), microglia (Iba1, green), and FGF13 (red). Scale bar = 100 μm. (N–O) Statistical analysis of the percentage of MAP2‐, GFAP‐, and Iba1‐positive cells frompanel M (N), and statistical analysis of the FGF13‐positive area percentage from panel M (O), *n* = 6 per group. (P) Astrocyte senescence model using D‐galactose (20 mg/mL, 48 h) induction in C6 cells. Representative fluorescence images showing GFAP (green) and FGF13 (red). SA‐β‐gal staining indicates astrocyte senescence levels. Scale bar = 100 μm. (Q–R) (Q) Representative western blots showing FGF13 protein changes and senescence markers p53 and p21 protein expression changes in aged C6 astrocytes. (R) Statistical analysis of FGF13, p53, and p21 protein expression levels from panel Q, *n* = 3 per group. All data are presented as the mean ± s.e.m. *p < 0.05, **p < 0.01.

To further investigate the impact of FGF13 on the nervous system during aging, we examined changes in neurons, astrocytes, and microglia. We found that neuron counts gradually decreased with aging (Figure 1M,N), while astrocytes and microglia gradually increased (Figure 1M,N). Meanwhile, cortical *FGF13* expression showed an initial increase followed by a decrease with age (Figure 1M,O). To determine if the role of FGF13 is specific, we constructed in vitro aging models of astrocytes and microglia. We found that FGF13 expression was significantly reduced in aging astrocytes (Figure 1P–R). Astrocytes provide nutritional and ionic support to neurons, regulate synaptic function, and participate in modulating microglial abnormal activation and inflammatory factor release. Based on this, we focused on investigating whether FGF13 exerts anti‐aging effects by regulating astrocytes.

### 3.2 Genetic Knockout of FGF13 in Astrocytes Exacerbates Aging‐Related Behaviors and the Aging Process

To explore the impact of FGF13 on aging progression, we first specifically knocked out *FGF13* in astrocytes within the mouse mPFC (FGF13‐KD). Adeno‐associated virus (AAV‐GFAP‐Cre) was injected into the mPFC of FGF13^fl/fl^ mice; details regarding virus injection and specificity are shown in Figure 2A,B. Further examination of *FGF13* expression levels confirmed efficient knockout (Figure 2C,D). Subsequently, the Morris water maze test was used to assess the effect of *FGF13* knockout on cognition and memory. The results showed that compared to young mice, the escape latency significantly increased in aging mice, and *FGF13* deficiency further increased this latency (Figure 2E,F). Given that each experimental group included equal numbers of male and female mice, we conducted additional analyses to assess whether sex influenced behavioral outcomes. As shown in Figure S2, no significant sex‐related differences in escape latency were observed within the Young, Aging, or FGF13‐KD groups, indicating that the effects of FGF13 manipulation on cognitive performance were independent of sex. In the memory test, the number of platform crossings and the time spent in the target quadrant were significantly reduced in both aging mice and FGF13‐KD mice (Figure 2G,H). In the subsequent open field test, the total distance moved significantly decreased in aging mice and FGF13‐KD mice compared to young mice (Figure 2I,J), as did the time spent in the center (Figure 2I,K). Furthermore, in the elevated plus maze test, the time spent in the open arms was significantly reduced in aging and FGF13‐KD mice compared to young mice (Figure 2L,M), and the number of entries into the open arms also significantly decreased (Figure 2L,N). These results indicate that *FGF13* knockout exacerbates behavioral and cognitive decline in aging mice.

**FIGURE 2:**
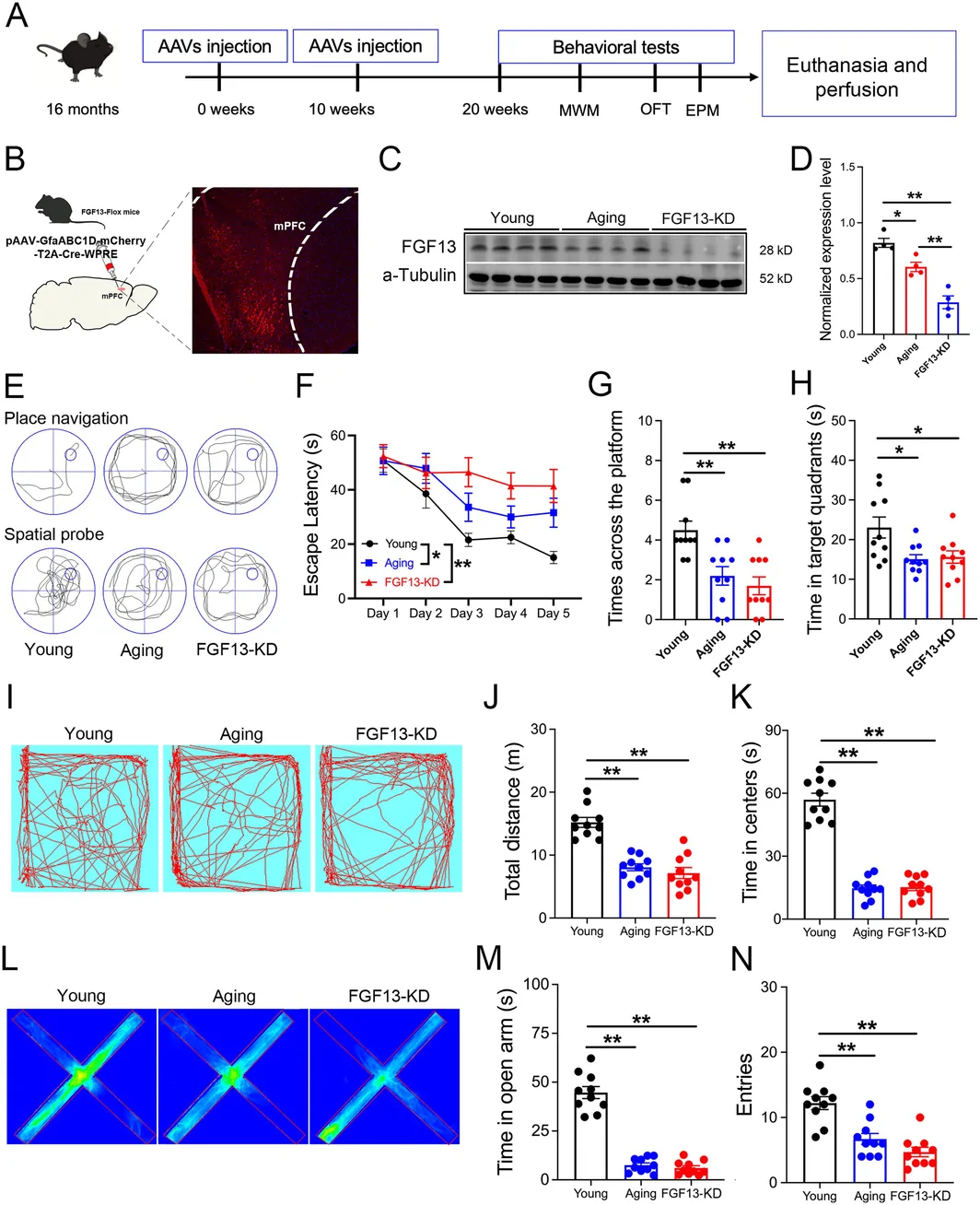
FGF13 knockout leads to cognitive decline in aged model mice. (A) Schematic diagram of the experimental timeline. Sixteen‐month‐old mice received viral injections every 10 weeks, and behavioral tests were conducted 20 weeks later. (B) Schematic of mouse strains, viral injection strategy, and representative image of viral expression in the mouse brain. Scale bar = 100 μm. (C–D) Detection and statistical analysis of FGF13 expression levels in the mPFC of FGF13 knockout model mice, *n* = 4 per group. (E–H) Morris water maze test: (E) Representative swimming trajectories of mice in each group. Statistical analysis of (F) escape latency during training, (G) number of platform crossings during the probe test 24 h later, and (H) time spent in the target quadrant during the probe test, *n* = 10 per group. (I–K) Open field test: (I) Representative movement trajectories of mice in each group. Statistical analysis of (J) total movement distance and (K) time spent in the center area, *n* = 10 per group. (L–N) Elevated plus maze test: (L) Heat maps representing mouse movement in each group. Statistical analysis of (M) time spent in the open arms and (N) number of entries into the open arms, *n* = 10 per group. All data are presented as the mean ± s.e.m. *p < 0.05, **p < 0.01.

To further investigate the effect of *FGF13* knockout on the aging process, we measured the levels of the senescence‐associated secretory phenotype (SASP) in mouse serum. We found that levels of pro‐inflammatory cytokines (TNF‐α, IFN‐γ, IL‐6, IL‐1β) were significantly elevated in the serum of aging mice compared to young mice (Figure 3A–D). Similarly, serum pro‐inflammatory cytokine levels in FGF13‐KD mice were significantly higher than in young mice (Figure 3A–D). Notably, the levels of TNF‐α and IL‐1β in FGF13‐KD mice were significantly higher than those in aging mice (Figure 3A,D). We also examined aging markers P53 and P21 in the mPFC and found that P53 levels were significantly higher in FGF13‐KD mice than in aging mice (Figure 3E,F). Additionally, we measured the levels of synaptic markers PSD95 and Synapsin‐1. The results showed that synaptic levels in the mPFC significantly decreased in aging and FGF13‐KD mice compared to young mice, with FGF13‐KD mice showing significantly lower levels than the aging group (Figure 3G,H). The brain is not only one of the earliest organs to age but may also serve as a “signaling hub” for systemic aging, amplifying and transmitting aging signals to other tissues via the blood to accelerate systemic aging. We examined aging levels in other organs (liver, spleen, kidney, heart, skin) and found that both aging mice and FGF13‐KD mice exhibited obvious signs of aging compared to young mice (Figure 3I–N), with FGF13‐KD mice displaying a more advanced aging state than aging mice. These results suggest that the loss of FGF13 in cortical astrocytes is a critical factor exacerbating aging.

**FIGURE 3:**
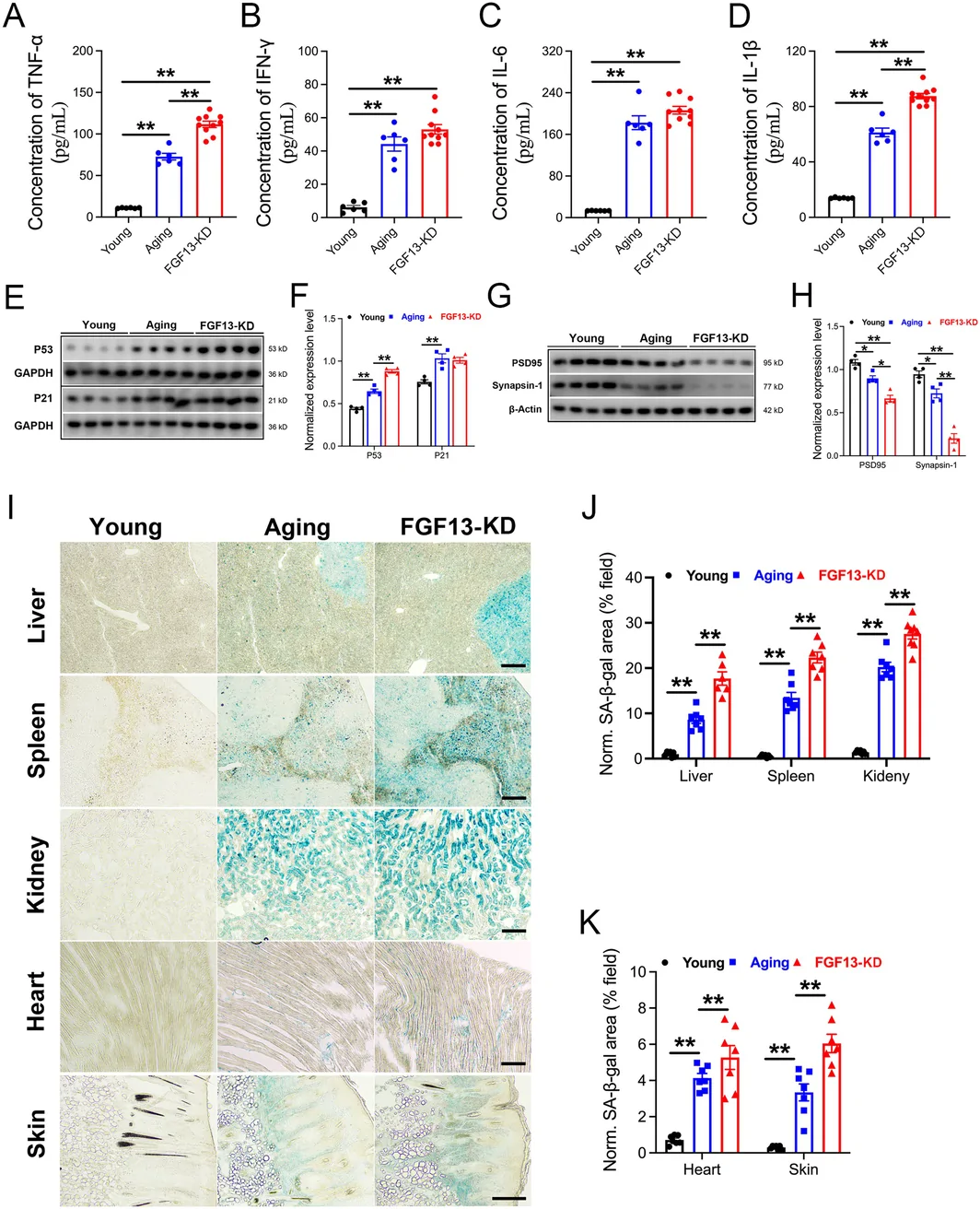
FGF13 deficiency exacerbates the aging process in mice. (A–D) Serum levels of pro‐inflammatory cytokines measured by ELISA in young, aged, and FGF13‐knockdown (FGF13‐KD) mice: TNF‐α (A), IFN‐γ (B), IL‐6 (C), and IL‐1β (D). *n* = 6–10 per group. (E–F) Representative western blots of aging markers P53 and P21 (E). Statistical analysis of P53 and P21 protein expression levels in the mPFC of young, aged, and FGF13‐KD mice (F). *n* = 4 per group. (G–H) Representative western blots of synaptic markers PSD95 and Synapsin‐1 (G). Statistical analysis of PSD95 and Synapsin‐1 protein expression levels in the mPFC of young, aged, and FGF13‐KD mice (H). *n* = 4 per group. (I–K) SA‐β‐gal staining showing senescence levels in the liver, spleen, kidney, heart, and skin of young, aged, and FGF13‐KD mice (I). Statistical analysis of senescence levels in the liver, spleen, and kidney (J), and in the heart and skin (K). *n* = 6–7 per group. All data are presented as the mean ± s.e.m. *p < 0.05, **p < 0.01.

### 3.3 Genetic Overexpression of FGF13 in Astrocytes Ameliorates Aging‐Related Behaviors and the Aging Process

To further investigate whether FGF13 can effectively ameliorate aging progression, we specifically overexpressed *FGF13* in astrocytes within the mouse mPFC (FGF13‐OE) by injecting adeno‐associated virus (AAV‐DIO‐FGF13) into the cortex of GFAP‐Cre mice (virus injection and specificity details in Figure 4A,B). Further testing showed that FGF13 expression levels were significantly higher than in aged mice (Figure 4C,D). Subsequently, the Morris water maze test was used to assess whether *FGF13* overexpression could improve cognition and memory. The results showed that compared to young mice, the escape latency significantly increased in aging mice, whereas *FGF13* overexpression effectively reduced the escape latency (Figure 4E,F). In the memory test, the number of platform crossings and time spent in the target quadrant significantly decreased in aging mice; however, these parameters significantly increased in the *FGF13* overexpression group compared to aging mice (Figure 4G,H). In the subsequent open field test, the total distance moved and time spent in the center significantly decreased in aging mice compared to young mice (Figure 4I–K); however, both total distance and time in the center were significantly higher in the *FGF13* overexpression group than in aging mice (Figure 4I,K). Furthermore, in the elevated plus maze test, while the time spent in open arms and the number of entries significantly decreased in aging mice compared to young mice (Figure 4L–N), the time spent in open arms was significantly greater in FGF13 overexpression mice than in aged mice (Figure 4L,M). These results indicate that FGF13 effectively improves behavior and cognition in aging mice.

**FIGURE 4:**
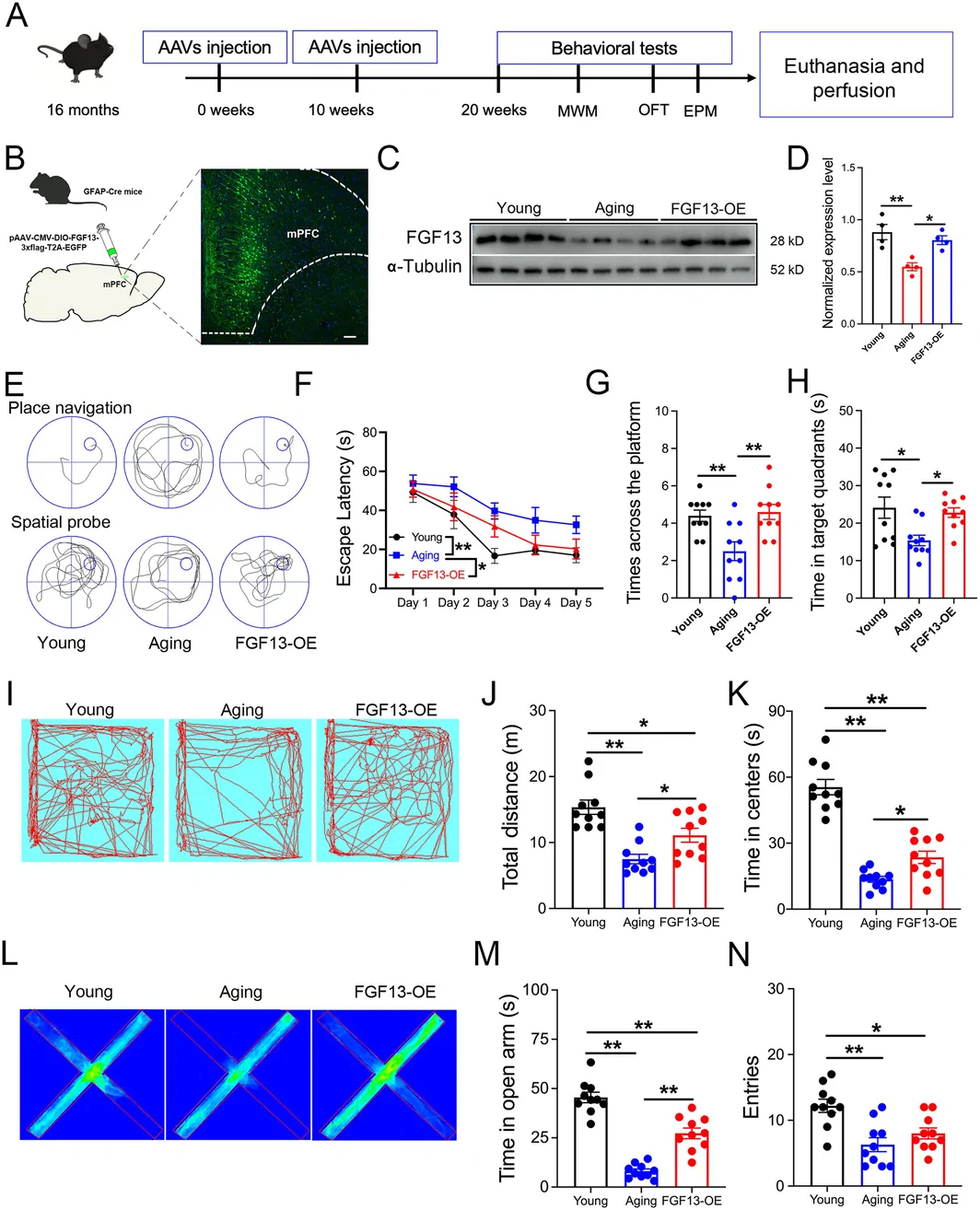
FGF13 ameliorates cognitive impairment and anxiety‐like behavior in aged model mice. (A) Schematic diagram of the experimental timeline. Sixteen‐month‐old mice received viral injections every 10 weeks, and behavioral tests were conducted 20 weeks later. (B) Schematic of mouse strains, viral injection strategy, and representative image of viral expression in the mouse brain. Scale bar = 100 μm. (C–D) Detection of FGF13 expression levels (C) and statistical analysis in the mPFC of FGF13 overexpression model mice (D), *n* = 4 per group. (E–H) Morris water maze test: (E) Representative swimming trajectories of mice in each group. Statistical analysis of (F) escape latency during training, (G) number of platform crossings during the probe test 24 h later, and (H) time spent in the target quadrant during the probe test, *n* = 10 per group. (I–K) Open field test: (I) Representative movement trajectories of mice in each group. Statistical analysis of (J) total movement distance and (K) time spent in the center area, *n* = 10 per group. (L–N) Elevated plus maze test: (L) Heat maps representing mouse movement in each group. Statistical analysis of (M) time spent in the open arms and (N) number of entries into the open arms, *n* = 10 per group. All data are presented as the mean ± s.e.m. *p < 0.05, **p < 0.01.

Furthermore, to investigate the effect of FGF13 on the aging process, we measured serum SASP levels. We found that relative to young mice, serum levels of pro‐inflammatory cytokines (TNF‐α, IFN‐γ, IL‐6, IL‐1β) were significantly elevated in aging mice (Figure 5A–D); however, FGF13 overexpression significantly reduced these levels in aging mice (Figure 5A–D). Simultaneously, we examined aging markers P53 and P21 in the mPFC and found that *FGF13* overexpression significantly reduced their protein expression levels in aging mice (Figure 5E,F). Additionally, we measured synaptic markers PSD95 and Synapsin‐1; relative to young mice, synaptic levels in the mPFC significantly decreased in aging mice, but *FGF13* overexpression significantly improved these levels (Figure 5G,H). We also examined aging levels in other organs (liver, spleen, kidney, heart, skin). While aging mice exhibited obvious signs of aging compared to young mice, *FGF13* overexpression significantly ameliorated aging levels across these organs (Figure 5I–N). These results indicate that overexpression of *FGF13* in cortical astrocytes effectively ameliorates aging in mice. Moreover, we also assessed the impact of FGF13 on body weight changes associated with aging in mice. Compared to the young group, aged mice displayed marked weight gain and an overt obese phenotype. Notably, FGF13 overexpression significantly attenuated this age‐related weight increase (Figure S3), implying that FGF13 may be involved in the regulation of lipid metabolism and inflammation during the aging process.

**FIGURE 5:**
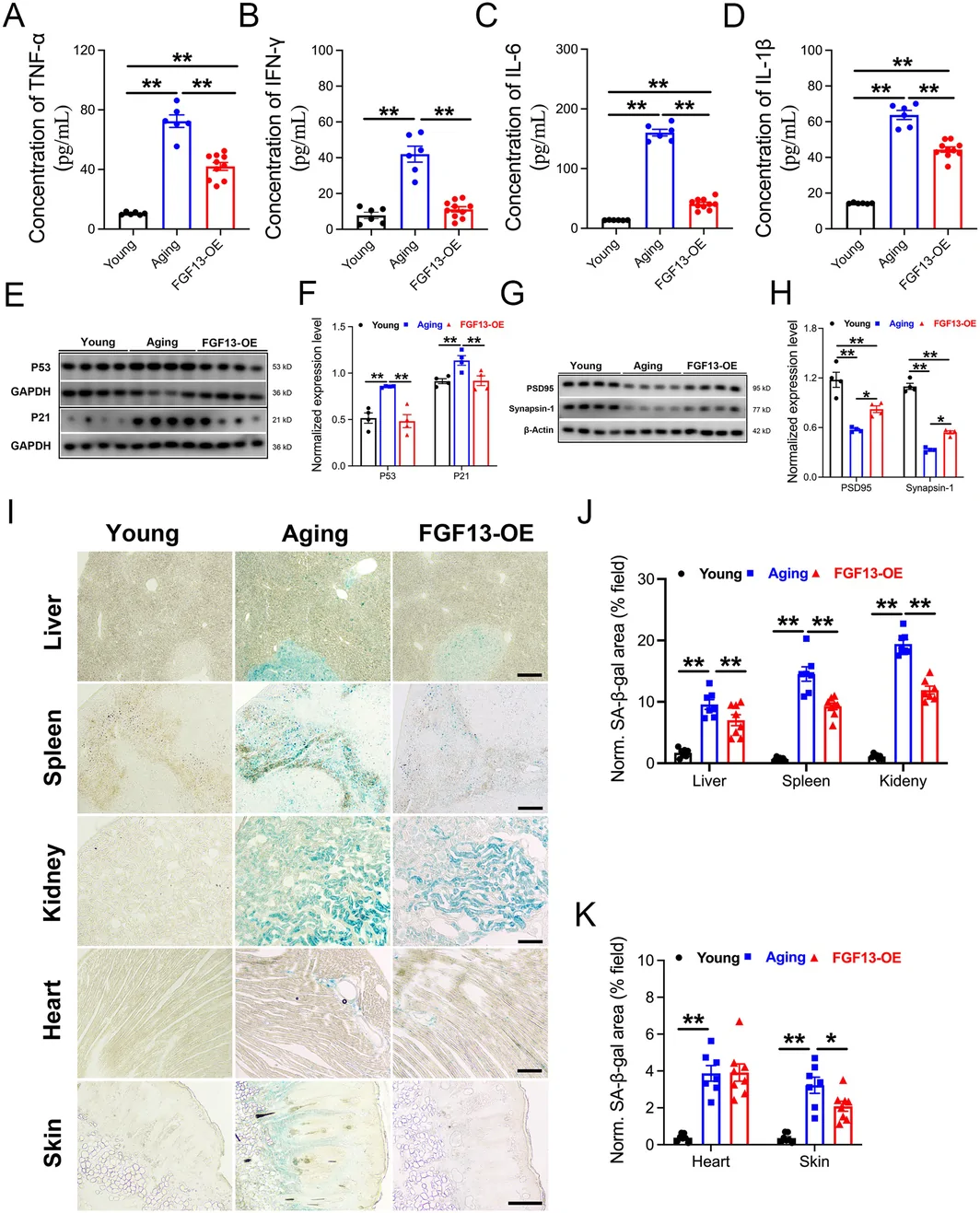
FGF13 alleviates the aging process in mice. (A–D) Serum levels of pro‐inflammatory cytokines measured by ELISA in young, aged, and FGF13‐overexpressing (FGF13‐OE) mice: TNF‐α (A), IFN‐γ (B), IL‐6 (C), and IL‐1β (D). *n* = 6–10 per group. (E–F) Representative western blots of aging markers P53 and P21 (E). Statistical analysis of P53 and P21 protein expression levels in the mPFC of young, aged, and FGF13‐OE mice (F). *n* = 4 per group. (G–H) Representative western blots of synaptic markers PSD95 and Synapsin‐1 (G). Statistical analysis of PSD95 and Synapsin‐1 protein expression levels in the mPFC of young, aged, and FGF13‐OE mice (H). *n* = 4 per group. (I–K) SA‐β‐gal staining showing senescence levels in the liver, spleen, kidney, heart, and skin of young, aged, and FGF13‐OE mice (I). Statistical analysis of senescence levels in the liver, spleen, and kidney (J), and in the heart and skin (K). *n* = 6–7 per group. All data are presented as the mean ± s.e.m. *p < 0.05, **p < 0.01.

### 3.4 FGF13 Deficiency Leads to Astrocyte Dysfunction and Impairs Neurological Function

To investigate the impact of FGF13 on the nervous system, we established an in vitro aging model using the C6 astrocyte cell line and knocked down FGF13 via shRNA. As shown in Figure 6A–D, FGF13 deficiency significantly exacerbated astrocyte senescence. Complement protein C3 is involved in maintaining brain development, relates to neurodegenerative diseases, and serves as a marker for toxic astrocytes (Chi et al. 2025). We found that FGF13 deficiency significantly increased C3 protein expression, and aging similarly led to elevated C3 levels (Figure 6C,D), suggesting that FGF13 deficiency exacerbates astrocyte neurotoxicity. Additionally, the gap junction protein connexin 43 (Cx43) plays a key role in maintaining CNS stability; it can induce excitatory amino acid transporter 2 (EAAT2) expression, accelerating glutamate release and leading to neuronal injury (Luo et al. 2011). We found that aging induced a significant increase in Cx43 expression in astrocytes, and FGF13 deficiency similarly led to a significant increase in Cx43 (Figure 6C,D). Concurrently, both FGF13 deficiency and aging significantly increased EAAT2 protein expression (Figure 6C,D). These results indicate that FGF13 deficiency induces astrocyte dysfunction.

**FIGURE 6:**
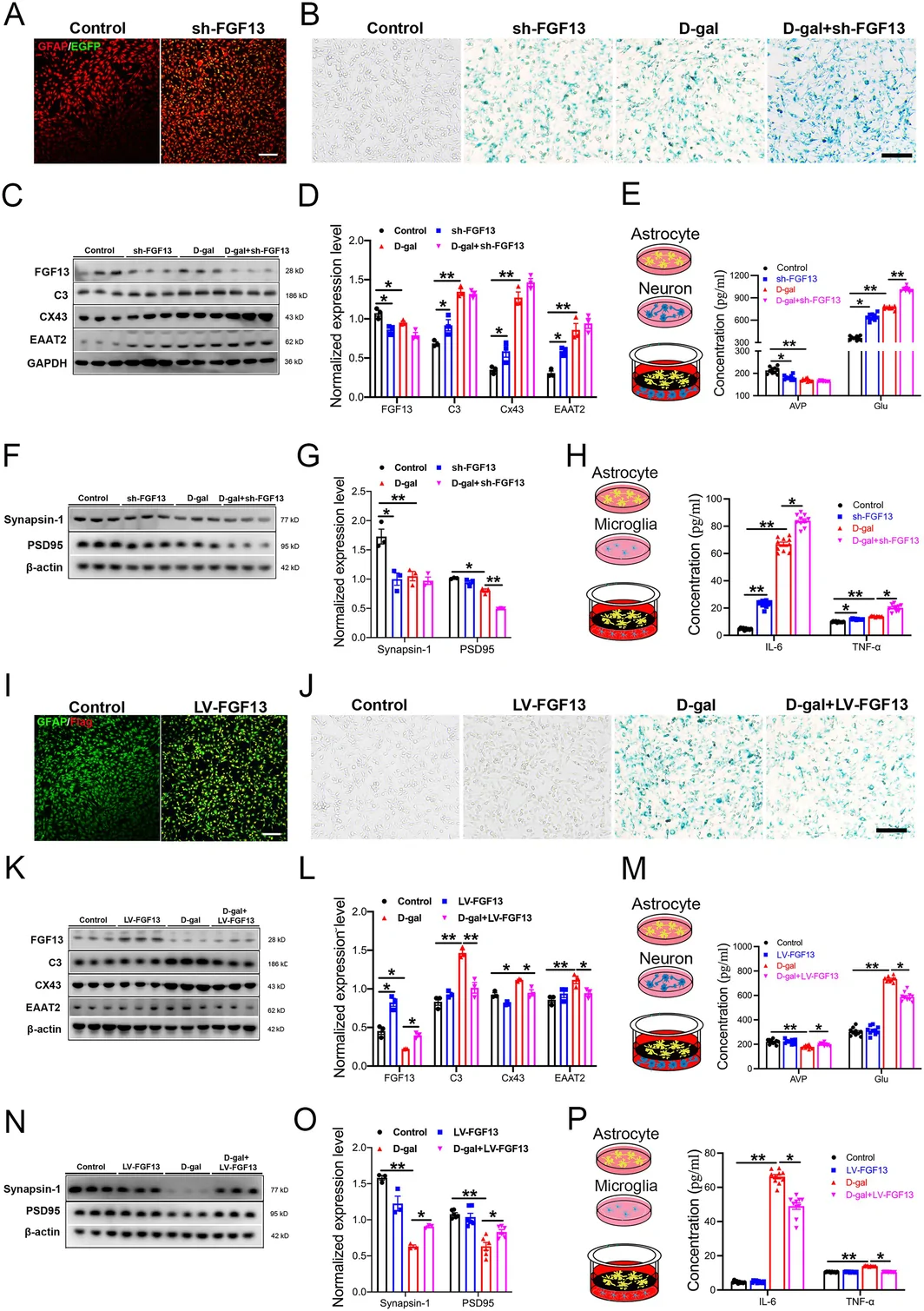
FGF13 modulates the function of senescent astrocytes. (A) Validation of the FGF13‐knockdown astrocyte model using sh‐RNA to interfere with FGF13 expression in C6 astrocytes. GFAP (red), EGFP (green). Scale bar = 100 μm. (B) Senescent astrocyte model induced by D‐galactose. SA‐β‐gal staining showing the effect of FGF13 knockdown on cellular senescence. Scale bar = 100 μm. (C–D) Representative western blots showing the effect of FGF13 knockdown on senescent astrocytes: Complement protein C3, connexin Cx43, and glutamate transporter EAAT2 (C). Statistical analysis of the protein expression levels (D), *n* = 3 per group. (E) (Left) Schematic of the co‐culture system of astrocytes (C6) and neurons (PC12). (Right) ELISA analysis showing the impact of senescent astrocytes on neurons: Levels of neuropeptide AVP and glutamate (Glu), *n* = 6 per group. (F–G) Representative western blots showing the impact of senescent astrocytes on neurons: Synaptic marker proteins Synapsin‐1 and PSD95 (F). Statistical analysis of Synapsin‐1 and PSD95 expression changes (G), *n* = 3 per group. (H) (Left) Schematic of the co‐culture system of astrocytes (C6) and microglia (BV2). (Right) ELISA analysis showing the impact of senescent astrocytes on microglia: Levels of inflammatory cytokines IL‐6 and TNF‐α, *n* = 6 per group. (I) Validation of the FGF13‐overexpression (LV‐FGF13) astrocyte model using pcSLenti‐CMV‐3xFlag‐PGK‐Puro‐WPRE3 to infect C6 astrocytes. GFAP (green), Flag (red). Scale bar = 100 μm. (J) Senescent astrocyte model induced by D‐galactose. SA‐β‐gal staining showing the effect of FGF13 overexpression on cellular senescence. Scale bar = 100 μm. (K–L) Representative western blots showing the effect of FGF13 overexpression on senescent astrocytes: Complement protein C3, connexin Cx43, and glutamate transporter EAAT2 (K). Statistical analysis of the protein expression levels (L), *n* = 3 per group. (M) (Left) Schematic of the co‐culture system of astrocytes (C6) and neurons (PC12). (Right) ELISA analysis showing the impact of senescent astrocytes on neurons: Levels of neuropeptide AVP and glutamate (Glu), *n* = 6 per group. (N–O) Representative western blots showing the impact of senescent astrocytes on neurons: Synaptic marker proteins Synapsin‐1 and PSD95 (N). Statistical analysis of Synapsin‐1 and PSD95 expression changes (O), *n* = 3 per group. (P) Schematic of the co‐culture system of astrocytes (C6) and microglia (BV2). ELISA analysis showing the impact of senescent astrocytes on microglia: Levels of IL‐6 and TNF‐α, *n* = 6 per group. All data are presented as the mean ± s.e.m. *p < 0.05, **p < 0.01.

To further explore the impact on the nervous system, we co‐cultured astrocytes (C6) with nerve cells (PC12). Results showed that both aging and FGF13 deficiency caused a significant decrease in neuropeptide (AVP) content and a significant increase in glutamate content in the culture medium (Figure 6E). Notably, FGF13 deficiency caused a significant increase in glutamate content produced by aging astrocytes (Figure 6E). We also detected synaptic proteins PSD95 and Synapsin‐1; results showed that both FGF13 deficiency and aging damaged neuronal synaptic structures (Figure 6F,G). Furthermore, in co‐cultures of astrocytes (C6) and microglia (BV2), FGF13‐deficient astrocytes stimulated microglia to release pro‐inflammatory factors IL‐6 and TNF‐α (Figure 6H). Aging astrocytes similarly stimulated this release (Figure 6H). Notably, FGF13 deficiency exacerbated the aging‐induced release of pro‐inflammatory factors (Figure 6H). In addition, the cross‐species co‐culture system (C6 and BV2) may introduce non‐specific xenogeneic immune responses, thereby affecting the accuracy of evaluating the release levels of inflammatory factors. To eliminate the potential confounding effects of cross‐species interactions, we established a same‐species co‐culture system using primary mouse astrocytes and BV2 microglia (both derived from mouse). Under D‐galactose‐induced senescence, senescent primary astrocytes showed significantly increased secretion of IL‐6 and TNF‐α. Furthermore, FGF13 knockdown exacerbated the release of these pro‐inflammatory cytokines, while FGF13 overexpression significantly attenuated their levels (Figure S4A–C). These results indicate that FGF13 deficiency leads to astrocyte dysfunction and further aggravates aging‐induced neurological impairment.

### 3.5 FGF13 Enhances Neurological Function by Ameliorating Aging‐Induced Astrocyte Dysfunction

To further investigate FGF13 regulation of astrocyte function during aging, we constructed an FGF13 overexpression viral vector and verified expression (Figure 6I,K,L). We found that overexpression of FGF13 effectively ameliorated D‐gal‐induced astrocyte senescence (Figure 6J). Further testing revealed that FGF13 effectively reduced the expression of the neurotoxic astrocyte marker C3 (Figure 6K,L) and significantly ameliorated the abnormally elevated levels of Cx43 and EAAT2 proteins caused by aging (Figure 6K,L). Additionally, in the neuron‐astrocyte co‐culture system, FGF13 effectively increased neuropeptide (AVP) content in the medium (Figure 6M) and reduced the abnormal elevation of glutamate caused by aging (Figure 6M). Examination of synaptic effects showed that FGF13 effectively increased the protein expression of PSD95 and Synapsin‐1 (Figure 6N,O), indicating effective amelioration of aging‐induced neuronal injury. Furthermore, in the astrocyte‐microglia co‐culture system, FGF13 effectively reduced the expression of pro‐inflammatory factors IL‐6 and TNF‐α (Figure 6P), indicating a reduction in aging‐induced inflammatory responses. These results demonstrate that FGF13 enhances neurological function by ameliorating aging‐induced astrocyte dysfunction.

### 3.6 FGF13 Participates in Regulating Astrocyte Inflammatory Responses and Autophagy

To investigate the mechanism of action, we performed RNA‐seq to determine how FGF13 ameliorates aging‐induced astrocyte dysfunction. Compared to the aging group, the FGF13 overexpression group showed 625 significantly upregulated genes and 195 significantly downregulated genes (Figure 7A). We performed GO and KEGG enrichment analyses on these genes. Upregulated genes were enriched in GO terms such as “Autophagosome‐lysosome fusion”, “Lysosomal transport”, “Growth factor activity”, “Signal transduction”, “Regulation of cytosolic calcium ion concentration”, “Glutamatergic synapse”, and “Glutathione transferase activity” (Figure 7B). KEGG analysis showed enrichment in “Autophagy – animal”, “Lysosome”, “Glutathione metabolism”, “PI3K‐Akt signaling pathway”, “Calcium signaling pathway”, “Axon guidance”, and “cAMP signaling pathway” (Figure 7C). Significantly downregulated genes were enriched in GO terms such as “Interleukin‐18 receptor activity”, “Negative regulation of gene expression”, “ATP hydrolysis activity”, “Cellular response to tumor necrosis factor”, and “Positive regulation of NF‐κB transcription factor activity” (Figure 7D). KEGG enrichment included “Cytokine‐cytokine receptor interaction”, “TGF‐beta signaling pathway”, “p53 signaling pathway”, “TNF signaling pathway”, and “cGMP‐PKG signaling pathway” (Figure 7E). In addition, we also conducted a GSEA analysis, showing a strong correlation between FGF13 and Autophagy (Figure 7F). These results indicate that FGF13 is primarily involved in regulating astrocyte autophagy and inflammatory responses.

**FIGURE 7:**
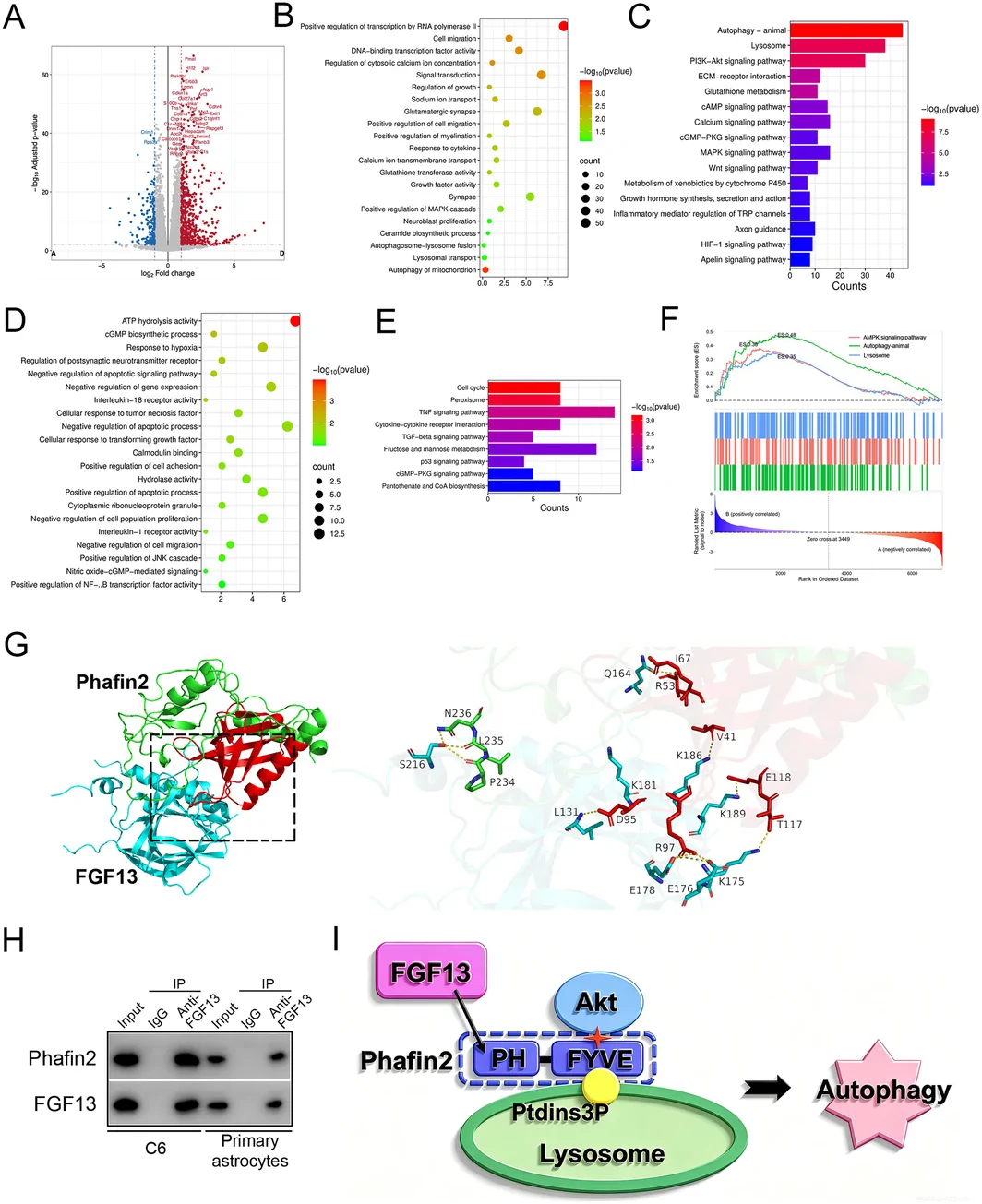
FGF13 modulates inflammatory response and autophagy in senescent astrocytes. (A) Volcano plot displaying the statistical analysis of RNA‐seq results from senescent astrocytes (control group) versus FGF13‐overexpressing senescent astrocytes. (B–C) GO enrichment analysis (B) and KEGG pathway enrichment analysis (C) of significantly upregulated differentially expressed genes following FGF13 overexpression. (D–E) GO enrichment analysis (D) and KEGG pathway enrichment analysis (E) of significantly downregulated differentially expressed genes following FGF13 overexpression. (F) GSEA analysis of Autophagy. (G) Molecular docking simulation analyzing the interaction between FGF13 and Phafin2. (H) Co‐immunoprecipitation (Co‐IP) assay detecting the interaction between FGF13 and Phafin2 in C6 cells and primary astrocytes. (I) Phafin2 is an elongated monomeric structure that contains an N‐terminal PH (Pleckstrin Homology) domain followed by a central FYVE domain. The PH domain is primarily involved in cellular signal transduction, while the FYVE domain is a small zinc‐binding module that, in most cases, specifically binds PtdIns3P and induces autophagy. Based on these findings, we speculate that FGF13 may bind to the PH domain of Phafin2, subsequently inducing Phafin2 activity and ultimately activating cellular autophagy.

### 3.7 FGF13 Regulates Astrocyte Autophagy by Targeting Phafin2

To further explore the biological mechanism, we integrated multiple protein interaction prediction platforms (STRING, Genemania, and HitPredict) and identified Phafin2 (PH domain‐containing family F member 2, Uniprot: Q9H8W4) as a potential direct target of FGF13. To verify this, we performed molecular docking simulations using AutoDock, which revealed a potential interaction between FGF13 and Phafin2 (Figure 7G). For further confirmation, we used Co‐IP and bimolecular fluorescence complementation assays, which revealed a specific interaction between FGF13 and Phafin2 (Figure 7H), providing important experimental evidence for the molecular mechanism.

Phafin2 is a phosphatidylinositol 3‐phosphate (PtdIns3P)‐binding effector involved in endosomal and lysosomal membrane‐associated signaling and in inducing autophagy (Tang et al. 2017). Phafin2‐mediated autophagy requires the presence of PtdIns(3)P on lysosomal membranes and the serine/threonine kinase Akt (Matsuda‐Lennikov et al. 2014). Furthermore, Phafin2 is an elongated monomeric structure that contains an N‐terminal PH (Pleckstrin Homology) domain followed by a central FYVE domain (Tang et al. 2017). The PH domain is primarily involved in cellular signal transduction, while the FYVE domain is a small zinc‐binding module that, in most cases, specifically binds PtdIns3P and induces autophagy (Matsuda‐Lennikov et al. 2014). Based on these findings, we speculate that FGF13 may bind to the PH domain of Phafin2, subsequently inducing Phafin2 activity and ultimately activating cellular autophagy (Figure 7I).

To further validate the above hypothesis, we next examined the effect of FGF13 on the Phafin2 signaling pathway. Our findings revealed that Phafin2 expression was significantly decreased in the mPFC of aged mice compared to that in young mice (Figure 8A,B), and the p‐Akt/Akt ratio was also markedly reduced. However, in FGF13‐overexpressing (FGF13‐OE) mice, the expression levels of Phafin2 and the p‐Akt/Akt ratio were significantly increased in the mPFC compared to those in aged mice (Figure 8A,B). To further investigate the effect of FGF13 on downstream autophagy, we found that markers LC3 were significantly reduced in the mPFC of aging mice relative to young mice (Figure 8A,B); however, FGF13 overexpression significantly increased the expression level of LC3 (Figure 8A,B). Furthermore, we found that the expression of the autophagy marker protein P62 significantly increased in the mPFC tissue of aging mice, while P62 significantly decreased in the brain tissue of FGF13‐OE mice (Figure 8A,B). Additionally, we examined cellular autophagy levels in the brain and found that while autophagosomes and autolysosomes decreased in aging mice, their numbers increased to some extent in the FGF13 overexpression group (Figure 8C). These results suggest that FGF13 improves cellular autophagy by activating the Akt via Phafin2.

**FIGURE 8:**
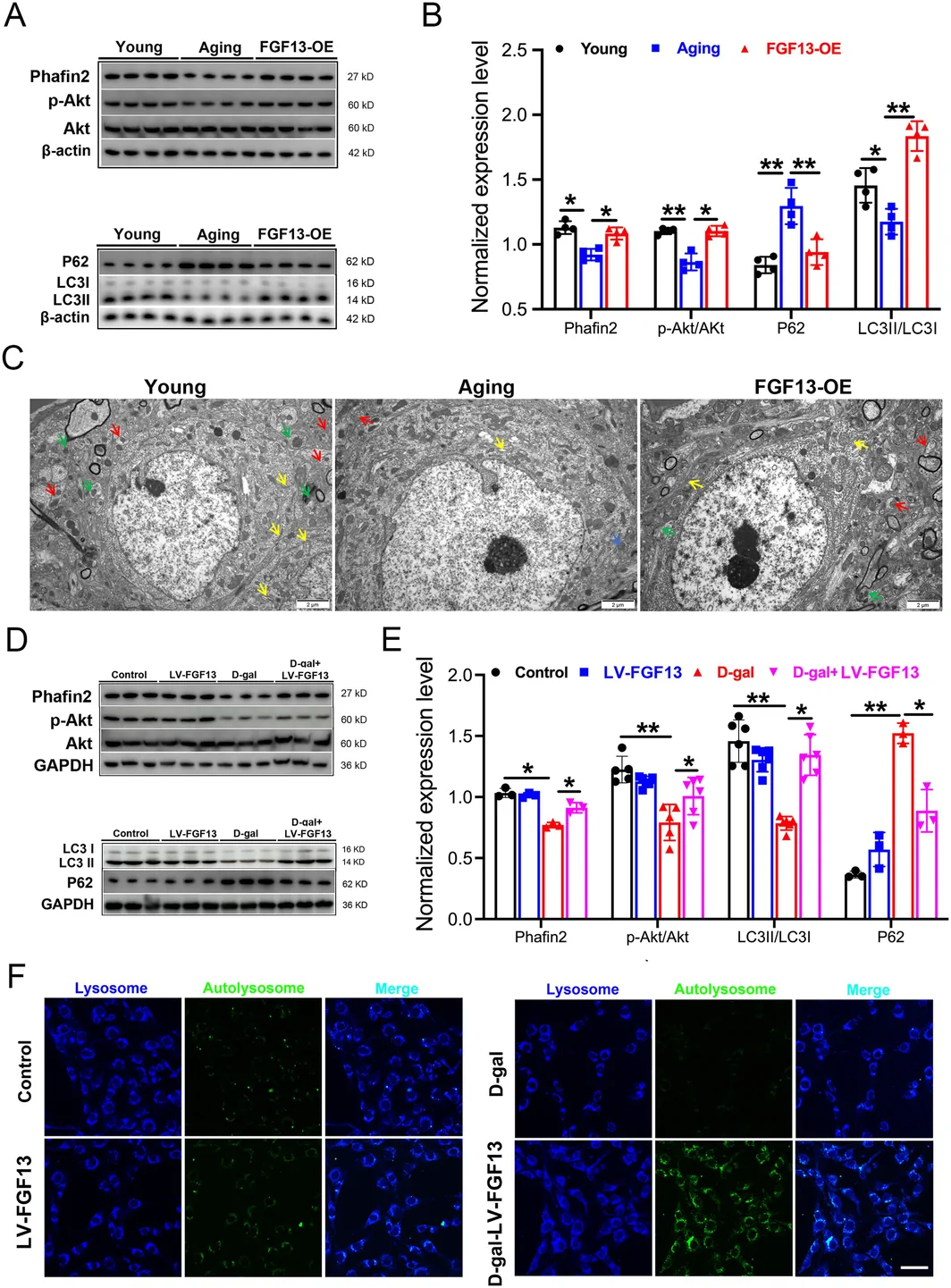
FGF13 regulates astrocyte autophagy via the Phafin2 signaling pathway. (A–B) Representative Western blots showing the effect of FGF13 overexpression on the Phafin2 signaling pathway (top) and downstream autophagy markers (bottom) in mouse cortical tissue (A). Statistical analysis of protein expression changes in the Phafin2 signaling pathway in young, aged, and FGF13‐overexpressing (FGF13‐OE) mice (B). *n* = 4 per group. (C) Transmission electron micrographs of cortical cells from young, aged, and FGF13‐OE mice, showing autophagy‐related structures: Autophagosomes (red arrows), autolysosomes (green arrows), phagophores (yellow arrows), and mitochondrial cristae dissolution/fragmentation (blue arrows). Scale bar = 1 μm. (D–E) Representative Western blots showing the effect of FGF13 overexpression (LV‐FGF13) on the Phafin2 signaling pathway (top) and downstream autophagy markers (bottom) in astrocytes (D). Statistical analysis of protein expression changes in the Phafin2 signaling pathway across experimental groups (E). *n* = 3 per group. (F) Representative fluorescence images showing changes in autophagy levels in astrocytes across groups: Lysosomes (blue), autolysosomes (green). Scale bar = 100 μm. All data are presented as the mean ± s.e.m. *p < 0.05, **p < 0.01.

To confirm these conclusions, we conducted in vitro cell experiments. We found that in the aging astrocyte model, Phafin2 expression was significantly reduced, but FGF13 overexpression (LV‐FGF13) significantly increased its protein levels (Figure 8D,E). Additionally, p‐Akt/Akt protein levels were significantly reduced in the aging astrocyte model, but FGF13 significantly increased the p‐Akt/Akt ratio in the aging astrocyte model (Figure 8D,E). We also found that the expression of autophagy markers LC3 was significantly decreased in aging astrocytes, but FGF13 significantly increased their levels (Figure 8D,E). Additionally, the expression of the autophagy marker protein P62 significantly increased in aging astrocytes, while overexpression of FGF13 could significantly inhibit the expression of P62 protein (Figure 8D,E). Using an autolysosome probe (which exhibits green fluorescence upon lysosome‐to‐autolysosome conversion), we found low autolysosome content in aging astrocytes, whereas FGF13‐overexpressing astrocytes showed increased autolysosome content (Figure 8F). To address the concern that C6 cells may exhibit transcriptional profiles distinct from those of primary astrocytes in vivo—which could potentially influence the functional outcomes of FGF13 modulation—we established primary astrocyte cultures derived from mouse cortex (Figure S4D) and assessed the effects of FGF13 on the Phafin2 signaling axis in these cells. We found that FGF13 overexpression significantly enhanced Phafin2 protein levels and increased the p‐Akt/Akt ratio, while concurrently reducing p62 expression and increasing the LC3II/LC3I ratio (Figure S4E,F). Collectively, these data demonstrate that FGF13 modulates autophagic flux in primary astrocytes via the Phafin2 pathway, fully recapitulating the results obtained in the C6 astrocytoma cell line.

## 4 Discussion

Aging is a complex biological process characterized by progressive deterioration of cellular and tissue homeostasis, yet the molecular mechanisms underlying region‐ and cell‐type‐specific vulnerability in the brain remain incompletely understood. (Hou et al. 2019; Mou et al. 2022). This study focuses on the relationship between brain tissue and neural cell aging. With increasing age, neural cells in the brain exhibit significant degenerative changes, with the rate of their aging and death far exceeding the regeneration process. This imbalance directly leads to memory decline and cognitive impairment. Notably, the degree of cell differentiation is positively correlated with vulnerability—the more specialized a cell's function, the more susceptible it is to damage. As the most highly differentiated cell type in the human body, neurons are particularly sensitive to aging, which explains why the brain is one of the earliest organs to show signs of aging (Butler et al. 2025; Bowirrat 2022). Single‐cell RNA sequencing enables rapid and efficient elucidation of mechanisms controlling complex systemic phenotypes such as aging (Hammond et al. 2019). Through single‐cell sequencing, we identified *FGF13* as a gene whose expression gradually declines with aging. The expression of FGF13 also varies across different neural cell types, with a notable downregulation observed particularly in astrocytes. Furthermore, regional susceptibility is a key characteristic of neurodegenerative diseases, though its impact on the aging process remains uncertain. Spatial transcriptomic analysis revealed a significant downregulation of *FGF13* expression in the cortical region, accompanied by a marked decrease in FGF13 protein levels. We therefore hypothesize that FGF13 deficiency may influence the aging process in mice by impairing cortical function.

FGF13 is a member of the intracellular FGF subfamily (FGF11–14) and, unlike canonical secreted FGFs, lacks a signal peptide and functions primarily in an intracrine manner without relying on FGFR activation (Prud'homme and Wang 2024; Kuro 2018). While other FGF family members, such as FGF21 and FGF23, have been implicated in systemic aging and metabolic regulation (Minami et al. 2024; Youm et al. 2025), the role of FGF13 in brain aging has remained largely unexplored. Notably, FGF13 has been identified as a candidate gene for X‐linked intellectual disability, and FGF13‐null mice exhibit impaired learning and memory (Yang et al. 2021). Some research suggests that FGF13 can exacerbate premature cardiac aging by regulating the Cav1‐p53‐p21 axis to increase cardiomyocyte senescence, whereas specific knockout of the *FGF13* gene in the heart ameliorates D‐galactose‐induced premature cardiac aging and functional impairment (Shen et al. 2025). However, other studies indicate that FGF13 can improve the aging process by regulating mitochondrial function and glucose homeostasis (Yang et al. 2021), while also preventing and treating metabolic diseases (Song et al. 2025; Wu et al. 2012). However, the cell‐type‐specific and region‐specific functions of FGF13 in the aging brain have not been systematically characterized. Our study addresses this gap by demonstrating that astrocytic FGF13 acts as a critical protective factor against aging‐related neuroinflammation and cognitive impairment.

Aging alters brain structure, and the mammalian nervous system is affected by aging in ways associated with molecular damage, potentially manifesting as cognitive and motor deficits that reduce quality of life (Omari et al. 2021). Astrocytes play a crucial role in the central nervous system (Clarke et al. 2018; Gildea and Liddelow 2025). In this study, we observed a significant age‐related decline in FGF13 expression in astrocytes. Concurrently, we found that the level of aberrant astrocyte activation in the brain significantly increases with age. Moreover, the serum levels of pro‐inflammatory factors in FGF13‐deficient mice were significantly higher than those in aging control mice, whereas FGF13 overexpression effectively reduced the inflammatory response. This finding resonates with the study by Allen WE et al. (Cell, 2023), which confirmed that the degree of astrocyte activation increases with age and that its spatial heterogeneity is closely related to local regional inflammation (Allen et al. 2023). Furthermore, astrocyte dysfunction can lead to neuronal dysfunction. In normal aging, individuals without overt neurological disease typically experience biological functional decline and cognitive deterioration. During aging, aberrantly activated astrocytes release massive amounts of inflammatory factors, inducing a cascade of pathological changes including endoplasmic reticulum (ER) stress, mitochondrial dysfunction, and impaired autophagy, ultimately accelerating cellular senescence. We found that FGF13 deficiency leads to dysfunction in C6 astrocytes, characterized by significantly elevated levels of C3 (a marker of neurotoxic astrocytes) and significant upregulation of EAAT2 (involved in glutamate transport). This not only causes nervous system injury but also exacerbates neuronal death. Additionally, Ling E et al. (Nature, 2024) (Ling et al. 2024) reported that significant impairments in neuronal synaptic function and astrocyte synaptic support (including adhesion, energy transfer, and neurotransmitter uptake) occur in the prefrontal cortex of the elderly, leading to reduced cognitive flexibility and neural plasticity. We detected synaptic damage in the mPFC of FGF13‐deficient mice, which was effectively ameliorated by FGF13 overexpression. Consistent results were obtained in an in vitro co‐culture system of aging astrocytes and neurons, where overexpression of FGF13 significantly reduced aging‐induced abnormal glutamate release, effectively protecting neurons. However, the inherent complexity and heterogeneity of the aging brain, where multiple parallel pathological mechanisms (neuroinflammation, synaptic loss, vascular dysfunction, etc.) converge and may partially buffer the effect of a single molecular intervention. But these results indicate that the downregulation of FGF13 expression is a key factor leading to abnormal astrocyte activation and functional impairment during aging, and that FGF13 may exert anti‐aging effects by regulating astrocyte function.

The biological mechanisms of FGF13 have been extensively studied. It has been found that FGF13 can activate the AKT‐GSK3 signaling pathway and inhibit the activity of p21 and p27 to participate in the regulation of tumor cell proliferation (Lu et al. 2020). Additionally, FGF13 participates in regulating NF‐κB signaling by targeting p65 to induce pathological cardiac hypertrophy and heart failure (Ivanov et al. 2011). Furthermore, FGF13 could block the release of damaged mitochondria by binding to the mitochondrial protein MCHT2 (a protein localized to the outer mitochondrial membrane), thereby inhibiting the activation of microglia and astrocytes and subsequently ameliorating neuroinflammation and neurodegeneration in mouse models of PD (Song et al. 2025). However, the mechanism of FGF13 in aging remains uncertain. Through transcriptome sequencing and targeted protein prediction, we discovered that FGF13 targets and binds to Phafin2. Phafin2 is a member of the Phafin protein family and participates in a wide range of cellular activities, including autophagy, endosomal trafficking, and micropinocytosis (Tang et al. 2017; Schink et al. 2021). Phafin2‐mediated autophagy requires Akt, and Phafin2 regulates autophagy by relocating cytoplasmic Akt to the autophagosome/autolysosome (Matsuda‐Lennikov et al. 2014). Autophagy not only actively degrades intracellular misfolded proteins or abnormal aggregates and clears superfluous, damaged, harmful, or ruptured organelles but also maintains the fine‐tuning of certain protein or lipid levels to ensure normal cellular function (Wang and Jia 2023). However, with increasing age, Akt activity gradually declines, leading to metabolic disturbances, oxidative stress, and increased inflammation (Kma and Baruah 2022; Xu, Han, et al. 2020). Simultaneously, the expression levels of autophagy‐related genes such as *ATG5*, *ATG7*, *ATG9*, and *BECN1* gradually decrease during aging (Xu, Li, et al. 2020). The decline in autophagosome‐lysosome fusion rates and impaired transport of autophagy substrates to lysosomes lead to a gradual reduction in cellular autophagy levels, ultimately resulting in the accumulation of metabolic waste in the brain and inducing neuroinflammation (Nixon 2024). Moreover, it was reported that increasing autophagy can effectively prevent premature aging and extend the lifespan of mice (Aman et al. 2021). Based on these findings, we propose that FGF13 promotes astrocyte autophagy by targeting the Phafin2 protein. By maintaining the functional stability of astrocytes and normal neuronal function, while also reducing microglia‐mediated inflammatory responses, FGF13 ameliorates the aging process in mice.

However, our study has several limitations that need to be considered. First, our RNA‐seq analysis and signaling pathway assessments were performed in the C6 astrocyte cell line, which may exhibit transcriptional profiles that differ from those of primary astrocytes and astrocytes in vivo. Second, Phafin2 contains PH and FYVE domains that enable it to bind PtdIns3P and Akt during autophagy, but whether FGF13 can directly bind to PtdIns3P or Akt remains unclear, as we were unable to investigate this further in the current study. Additionally, our in vivo behavioral analyses revealed that FGF13 knockdown in aged mice did not further exacerbate cognitive deficits beyond the already pronounced age‐related impairment, likely due to a behavioral floor effect in 20–23‐month‐old mice. This does not exclude the possibility that FGF13 loss may produce measurable deficits in young adult mice, where baseline performance is not yet compressed. In addition, our control design, while including age‐matched FGF13^fl/fl^ and GFAP‐Cre mice, did not independently rule out potential Cre toxicity or floxed allele effects in the aged context. Although our previous work (Zheng et al. 2026) has shown no effects of these elements on behavior in young mice, their impact in aging remains to be formally tested. Future studies may need to employ more refined assessment methods to address these issues.

In summary, during early aging, the prefrontal cortex gradually undergoes atrophy due to chronic inflammatory injury, leading to cognitive and memory impairment. The inflammatory response mediated by sustained abnormal activation of astrocytes is a critical factor inducing long‐term chronic inflammation, and the significant downregulation of FGF13 expression in astrocytes is a major cause of this abnormal activation. Our study reveals the function of FGF13 in astrocytes and elucidates its anti‐aging mechanism: targeting Phafin2 to activate cellular autophagy to maintain astrocyte homeostasis. This study not only provides a new theoretical perspective for research on the functions of the FGF family and deepens our understanding of aging mechanisms but may also help uncover novel anti‐aging therapeutic targets, offering innovative strategies for promoting healthy aging.

## Author Contributions

All authors contributed to the study design. Rui Zheng, Zheng Tu, Yang Fang and Caixiang Zhuang analyzed the data, the data collection was performed by Rui Zheng, Zheng Tu, Caixiang Zhuang, Yuchao Zhang, Yibo Ying, Ziyang Jin and Zhiyi Feng. The first draft of the manuscript was written by Zhouguang Wang, Yanyan Zheng, and Rui Zheng. Yanyan Zheng and Zhouguang Wang helped to revise the manuscript. All authors read and approved the final manuscript.

## Funding

This work was supported by Wenzhou Municipal Science and Technology Bureau (Y20240311) and Young Scientists Fund (B) of the National Natural Science Foundation of China (82522081).

## Ethics Statement

All animal studies have been approved by Animal Care Ethics Guidelines of Wenzhou Medical University and were approved by the Ethics Committee (XMSQ2025‐0259) and were in accordance with the ARRIVE guidelines on the Care and Use of Experimental Animals.

## Conflicts of Interest

The authors declare no conflicts of interest.

## Supporting information


**Table S1:** Primary Antibody Information.
**Figure S1:** Information of the publicly GEO datasets used in this study. A. Public GEO datasets from aging‐related models. B–C. Spatial transcriptomic sequencing data from aged mouse brain tissue (public database GSE193107). (B) Expression distribution of the FGF13 gene across different brain regions. (C) Statistical analysis of FGF13 expression differences in the cortical region. D. Cell type composition in the GSE129788 dataset. E–J. Cell counts in young versus aged mice: neurons (E), astrocytes (F), microglia (G), oligodendrocytes (H), OPCs (I), and DCs (J). K–O. FGF13 expression levels in young versus aged mice across cell types: neurons (K), astrocytes (L), microglia (M), oligodendrocytes (N), and DCs (O).
**Figure S2:** Analysis of sex‐related behavioral differences in the Morris water maze. A–B. Swimming speed was analyzed across all experimental groups (A–B), *n* = 10–12 mice per group. C–F. Escape latency was compared between male and female mice within each group, including the young (C), aging (D), FGF13‐KD (E), and FGF13‐OE (F) groups, *n* = 5–6 mice per group.
**Figure S3:** Effect of FGF13 on body weight in aged mice. (A) Physical appearance of young, aged, FGF13‐knockdown (FGF13‐KD), and FGF13‐overexpressing (FGF13‐OE) mice: female (left), male (right). (B) Body weight changes over time in each group, *n* = 10 per group. All data are presented as the mean ± s.e.m. *p < 0.05, **p < 0.01.
**Figure S4:** FGF13 attenuates aging‐related changes in primary astrocytes. A. Schematic of the primary astrocyte–BV2 microglia co‐culture system. B–C. *ELISA* quantification of IL‐6 (B) and TNF‐α (C) secretion *n* = 6/group. D. Immunofluorescence staining of primary astrocytes showing GFAP (green), Flag (red), and DAPI (blue). Scale bar = 50 μm. E–F. Western blot analysis of the Phafin2 signaling pathway: representative blots (E) and densitometric quantification (F). All data are presented as the mean ± s.e.m. *p < 0.05, **p < 0.01.

## Data Availability

The data that support the findings of this study are available on request from the corresponding author. The data are not publicly available due to privacy or ethical restrictions.
