# Supplementary material for: FGF13 Alleviates the Aging Process in Mice by Regulating Astrocyte Function in the Medial Prefrontal Cortex via Phafin2 Signaling Pathway
Source: Aging Cell. 2026 Oct 25;25(11):e70784. doi: 10.1111/acel.70784 (PMC13749145; doi:10.1111/acel.70784)
Supplement: Supplementary file 1 [file ACEL-25-e70784-s001.docx]

**Supplementary Data**

**Table S1. Primary Antibody Information**

| Name | Code | Producer |
| --- | --- | --- |
| FGF13 | MA5-27705 | Invitrogen |
| C3 | PA5-21349 | Invitrogen |
| P21 | A11454 | Abclonal |
| P53 | 10442-1- AP | Proteintech |
| GAPDH | 60004-1-Ig | Proteintech |
| Connexin 43 | ab314908 | Abcam |
| LC3 | ab192890 | Abcam |
| MAP2 | ab318993 | Abcam |
| GFAP | ab7260 | Abcam |
| Iba-1 | ab178846 | Abcam |
| Synaptophysin | ab32127 | Abcam |
| P62 | ab109012 | Abcam |
| alpha-Tubulin | 2144S | Cell Signaling Technology |
| beta-actin | 4967S | Cell Signaling Technology |
| Akt | 9272S | Cell Signaling Technology |
| p-Akt | 4060T | Cell Signaling Technology |
| PSD95 | 2507S | Cell Signaling Technology |
| EAAT2 | 3838S | Cell Signaling Technology |
| Phafin2 | HPA024829 | Sigma |

**Figure S1. Information of the publicly GEO datasets used in this study.**

A. Public GEO datasets from aging-related models.

B–C. Spatial transcriptomic sequencing data from aged mouse brain tissue (public database GSE193107). (B) Expression distribution of the FGF13 gene across different brain regions. (C) Statistical analysis of FGF13 expression differences in the cortical region.

D. Cell type composition in the GSE129788 dataset.

E–J. Cell counts in young versus aged mice: neurons (E), astrocytes (F), microglia (G), oligodendrocytes (H), OPCs (I), and DCs (J).

K–O. FGF13 expression levels in young versus aged mice across cell types: neurons (K), astrocytes (L), microglia (M), oligodendrocytes (N), and DCs (O).

**Figure S2.** Analysis of sex-related behavioral differences in the Morris water maze.

A-B. Swimming speed was analyzed across all experimental groups (A–B), n =10–12 mice per group.

C-F. Escape latency was compared between male and female mice within each group, including the young (C), aging (D), FGF13-KD (E), and FGF13-OE (F) groups, n = 5-6 mice per group.

**Figure S3.** Effect of FGF13 on body weight in aged mice. (A) Physical appearance of young, aged, FGF13-knockdown (FGF13-KD), and FGF13-overexpressing (FGF13-OE) mice: female (left), male (right). (B) Body weight changes over time in each group, n = 10 per group. All data are presented as the mean ± s.e.m. *p < 0.05, **p < 0.01.

**Figure S4. FGF13 attenuates aging-related changes in primary astrocytes.**

A. Schematic of the primary astrocyte–BV2 microglia co-culture system.

B–C. ELISA quantification of IL-6 (B) and TNF-α (C) secretion n = 6/group.

D. Immunofluorescence staining of primary astrocytes showing GFAP (green), Flag (red), and DAPI (blue). Scale bar = 50 μm.

E–F. Western blot analysis of the Phafin2 signaling pathway: representative blots (E) and densitometric quantification (F). All data are presented as the mean ± s.e.m. *p < 0.05, **p < 0.01.
